# Characteristics predicting the efficacy of SGLT-2 inhibitors versus GLP-1 receptor agonists on major adverse cardiovascular events in type 2 diabetes mellitus: a meta-analysis study

**DOI:** 10.1186/s12933-023-01877-6

**Published:** 2023-06-28

**Authors:** Minji Sohn, Johannes W. Dietrich, Michael A. Nauck, Soo Lim

**Affiliations:** 1 Department of Internal Medicine, Seoul National University Bundang Hospital, Seoul National University College of Medicine, 82, Gumi-ro 173 Beon-gil, Bundang-gu, Seongnam, 13620 Republic of Korea; 2 Diabetes, Endocrinology and Metabolism Section, Department of Medicine I, St. Josef-Hospital (Ruhr-University Bochum), Gudrunstr. 56, D-44791 Bochum, Germany

**Keywords:** Sodium–glucose cotransporter-2 inhibitor, Glucagon-like peptide 1 receptor agonist, Major adverse cardiovascular events, Meta-regression, Meta-analysis, Diabetes mellitus, type 2

## Abstract

**Background:**

Recent large clinical trials have demonstrated cardiovascular benefits of similar overall magnitude for sodium–glucose cotransporter-2 inhibitor (SGLT-2i) and glucagon-like peptide-1 receptor agonist (GLP-1RA) therapy in subjects with type 2 diabetes. We sought to identify subgroups based on baseline characteristics with a differential response to either SGLT-2i or GLP-1RA.

**Methods:**

PubMed, Cochrane CENTRAL, and EMBASE were searched from 2008 to 2022 for SGLT-2i or GLP-1RA randomized trials that reported 3-point major adverse cardiovascular events (3P-MACE). Baseline clinical and biochemical characteristics included age, sex, body mass index (BMI), HbA1c, estimated glomerular filtration rate (eGFR), albuminuria, preexisting cardiovascular disease (CVD), and heart failure (HF). Absolute and relative risk reductions (ARR and RRR) regarding incidence rates for 3P-MACE with a 95% confidence interval were calculated. The association of average baseline characteristics in each study with the ARR and RRR for 3P-MACE was investigated by meta-regression analyses (random-effects model, assuming inter-study heterogeneity). Meta-analysis was also conducted to investigate whether the efficacy of SGLT-2i or GLP-1RA on 3P-MACE reduction could differ according to the patient’s characteristics (e.g., HbA1c above/below cutoff).

**Results:**

After a critical assessment of 1,172 articles, 13 cardiovascular outcome trials with a total of 111,565 participants were selected. In meta-regression analysis, the more patients with reduced eGFR in the studies, the greater ARR by SGLT-2i or GLP-1RA therapy. Similarly, in the meta-analysis, SGLT-2i therapy tended to be more effective in reducing 3P-MACE in people with eGFR < 60 ml/min/1.73 m^2^ than in those with normal renal function (ARR − 0.90 [–1.44 to − 0.37] vs. − 0.17 [–0.34 to − 0.01] events/100 person-years). Furthermore, people with albuminuria tended to respond better to SGLT-2i therapy than those with normoalbuminuria. However, this was not the case for the GLP-1RA treatment. Other factors including age, sex, BMI, HbA1c, and preexisting CVD or HF did not affect the efficacy of either SGLT-2i or GLP-1RA treatment on the ARR or RRR of 3P-MACE.

**Conclusions:**

Because decreased eGFR [significant] and albuminuria [trend] were found to predict a better efficacy for SGLT-2i in 3P-MACE reduction, this class of drug should be preferred in such patients. However, GLP-1RA may be considered for patients with normal eGFR because it showed better efficacy than SGLT-2i in this subgroup [trend].

**Supplementary Information:**

The online version contains supplementary material available at 10.1186/s12933-023-01877-6.

## Background

Diabetes mellitus (DM) is one of the major causes of death in humans and doubles the risk of cardiovascular disease (CVD) in the United States (US) [1]. The 2008 US Food and Drug Administration antidiabetic drug guidance mandated that novel antihyperglycemic medications should demonstrate cardiovascular safety through large cardiovascular outcome trials (CVOTs) [2]. Accordingly, CVOTs comparing dipeptidyl peptidase-4 inhibitors, sodium–glucose cotransporter-2 inhibitors (SGLT-2is), and glucagon-like peptide-1 receptor agonists (GLP-1RAs) with placebo on a background of standard of care have been conducted [3, 4]. Among them, certain SGLT-2i and GLP-1RA compounds have shown not only safety but superiority in their effects on cardiovascular outcomes [5].

Of note, there are some concerns about adverse events in SGLT-2i and GLP-1RA therapy in clinical practice. In the early days, many doctors were reluctant to use SGLT-2i in patients with reduced renal function or older patients, particularly because of concerns about adverse effects on the kidney. However, recent trials with SGLT-2i in patients with chronic kidney disease have proven significant efficacy on composite renal outcomes [6–8]. Moreover, there was no difference in the cardiovascular benefits from SGLT-2i therapy between younger and older patients [9]. Moreover, SGLT-2i therapy seemed to be more effective regarding composite cardiovascular outcomes in patients with preexisting CVD [10, 11].

Physicians tend to avoid prescribing GLP-1RA to old people and those with low body mass index (BMI) because of its gastrointestinal adverse events [12]. However, more evidence is needed to support this practice pattern. On the contrary, it was speculated that people with overweight or obesity respond well to GLP-1RA because the therapy can reduce weight. In addition, some GLP-1RA types, such as dulaglutide and liraglutide, showed benefits in composite renal outcomes in the CVOTs [13, 14]. However, distinctive beneficial effects of GLP-1RA on cardiovascular outcomes in patients with obesity or those with renal impairment vs. those without these conditions have not been established yet.

Taken together, there is a clinical interest in determining patient factors that predict a differential therapeutic response to SGLT-2i and GLP-1RA (in the sense that subgroups are identified who respond better to one and worse to the alternative treatment).

To the best of our knowledge, no systematic approach has ever been taken to identify the patient characteristics related to the effectiveness of these two agents. Therefore, the current study was designed to compare the 3-point major adverse cardiovascular event (3P-MACE) risk reduction with SGLT-2i and GLP-1RA in subgroups of patients participating in the large CVOTs. Through this approach, we aimed to suggest recommendations for individualizing the choice between these two cardioprotective medications in patients with type 2 DM (T2DM).

## Methods

### Data sources and study selection

We conducted a systematic review and meta-analysis following the updated guidance in the Preferred Reporting Items for Systematic Reviews and Meta-Analysis (PRISMA 2020) [15]. Neither ethics approval nor patient consent was required for this analysis. The review was registered in PROSPERO (CRD42021235989).

Databases searched included MEDLINE, EMBASE, and the Cochrane Central Register of Controlled Trials. The latest searches were conducted in December 2022, focusing on the period from January 2008 to December 2022. The search terms included the following keywords: ‘type 2 diabetes’ for the population; ‘sodium-glucose cotransporter 2 inhibitor’ and ‘glucagon-like peptide-1 receptor agonist’ (or the individual compounds in these classes) for the study intervention; ‘major adverse cardiovascular event’ and ‘cardiovascular event’ for outcomes. Searches were restricted to clinical trials and those reported in the English language.

We included trials using the following inclusion criteria: (1) evaluation of an antidiabetic agent compared with placebo; (2) report of 3P-MACE for the overall population and subgroups (grouped by patient characteristics); (3) a minimum number of 1,000 T2DM subjects enrolled; and (4) multinational randomized controlled trials (RCTs) with a wide representation of ethnic backgrounds.

### Outcomes

The main outcomes were the relative and absolute risk reductions (RRR and ARR) for 3P-MACE by subgroup (based on patient characteristics presented at baseline) comparing SGLT-2i and GLP-1RA treatment, aiming to identify differential responses. The baseline characteristics included age, sex, BMI, initial HbA1c level, baseline renal function (estimated glomerular filtration rate, eGFR), presence of albuminuria, and preexisting CVD or heart failure (HF): 3P-MACE combined cardiovascular death, nonfatal myocardial infarction, and nonfatal stroke.

### Data extraction

Data were extracted independently by two authors (M.S. and S.L.). The following data were extracted for the included studies: name of the trial, year of publication, number of patients, patient characteristics, comorbidity status, and concurrent medication. To check the population risks for CVDs, event rates of 3P-MACE were collected from each trial. The outcomes included the event rate, event number, and sample size of the group.

### Data synthesis and analysis

Data were analyzed using R software (version 4.1.0; R Development Core Team, Vienna, Austria) with the ‘metafor’ package. Included trials were assessed for the risk of bias using the Cochrane risk of bias 2 tool. The *I*^*2*^ statistic was used to assess the overall heterogeneity of all the comparisons, with values of under 25%, 50%, and 75% corresponding to mild, moderate, and high heterogeneity, respectively.

ARR and RRR were calculated using the number of events and patient-years of observation [16], and then used for pooled estimates and their 95% confidence intervals (CIs). If these data were not explicitly reported, they were approximated: event rate = [(number of people with the event [n]) / ([total person-years of observation] × 100]).

We performed a meta-regression analysis of the selected trials to assess the relationship between the patient characteristics at baseline (based on mean values, e.g., for BMI, or proportions with a characteristic of interest, e.g., patients with preexisting CVD) and the corresponding ARR and RRR for 3P-MACE. We also estimated the strength of the association of the characteristics by estimating R^2^. The ‘rma’ function was used to examine the effects of drug types as moderators. The outcomes among different baseline subgroups or therapies were compared using frequentist meta-analysis with random-effects models.

### Quality of evidence

The Grading of Recommendations Assessment, Development and Evaluation (GRADE) method was used to assess the quality and strength of the evidence for each subgroup. Two authors (M.S. and S.L.) rated the quality of the evidence for each outcome independently. We used GRADEpro software (McMaster University and Evidence Prime Inc., Hamilton, Ontario, Canada) to generate evidence profile tables. An *I*^*2*^ value over 50% was regarded as an indication of serious inconsistency. Imprecision was assessed as serious if the reported subgroups were less than half and as very serious when only one trial reported the results. In terms of 3P-MACE, none of the meta-analyses were considered to have serious indirectness.

## Results

### Results of the search and study characteristics

We identified 1,172 articles. After critically assessing these papers, 13 CVOTs (6 SGLT-2i trials and 7 GLP-1RA trials) fulfilled the inclusion criteria (Supplementary Figure S1), representing 111,565 participants. However, the ELIXA trial was excluded because subgroup data for 3P-MACE were not reported.

The characteristics of the included trials and the recruited patients are presented in Table 1. The mean follow-up time range was 1.0–5.5 years. The mean age range of participants was 62–69 years, and the proportion of men varied between 53.7% and 71.5%. The percentage of patients with preexisting CVD ranged from 31.5 to 100%. The eGFR < 60 ml/min/1.73 m^2^ at baseline ranged from 7.4 to 100%. The 3P-MACE event rates in individual studies were 2.4–6.8 per 100 person-years with placebo and 2.3–5.4 per 100 person-years with active treatment.

**Table 1:** Baseline Characteristics of Cardiovascular Outcome Trials with SGLT-2is or GLP-1RAs in Patients with Type 2 Diabetes

Trial	Mean Follow-up	N	Participants’ characteristics		Characteristics of diabetes	Characteristics of comorbidities		Concurrent medication		Risk of events*
CANVAS program [25] (Canagliflozin)	3.6 yr	10,142	Age: 63.3 yr Men: 64.2%	BMI: 32 kg/m^2^ SBP: 136.6 mmHg LDLc: 2.3 mmol/L	HbA1c: 8.2% DM duration: 13.5 yr	HTN: 90.0% CVD: 65.6% HF: 14.4%	eGFR < 60: 20.1% ACR ≥ 30 mg/g: 29.8%		Statin 74.9% Antithrombotics 73.6% RAAS blockers 80.0%	3P-MACE 2.7/3.2 (100PY)
CREDENCE [35] (Canagliflozin)	2.6 yr	4,401	Age: 63 yr Men: 66.1%	BMI: 31.3 kg/m^2^ SBP: 140.0 mmHg LDLc: 2.5 mmol/L	HbA1c: 8.3% DM duration: 15.8 yr	HTN: 96.8% CVD: 50.4% HF: 14.8%	eGFR < 60: 59.8% ACR ≥ 30 mg/g: 99.3%		Statin 69.0% Antithrombotics 59.6% RAAS blockers 99.9%	3P-MACE 3.9/4.9 (100PY)
DECLARE-TIMI 58 [36] (Dapagliflozin)	4.2 yr†	17,160	Age: 63.9 yr Men: 62.6%	BMI: 32.1 kg/m^2^ SBP: 135.8 mmHg LDLc: 2.3 mmol/L	HbA1c: 8.3% DM duration: 11.8 yr	HTN‡: 89.4% CVD: 40.6% HF: 10.0%	eGFR < 60: 7.4% ACR ≥ 30 mg/g: 30.3%		Statin 75.0% Antithrombotics 61.1% RAAS blockers 81.3%	3P-MACE 2.3/2.4 (100PY)
EMPA-REG OUTCOME [11] (Empagliflozin)	3.0 yr	7,020	Age: 63.1 yr Men: 71.5%	BMI: 30.6 kg/m^2^ SBP: 135.5 mmHg LDLc: 2.2 mmol/L	HbA1c: 8.1% DM duration > 10 year: 57.1%	HTN‡: 94.6% CVD: 100% HF: 10.1%	eGFR < 60: 25.9% ACR ≥ 30 mg/g: 39.6%		Statin 77.0% Antithrombotics 89.1% RAAS blockers 80.7%	3P-MACE 3.7/4.4 (100PY)
VERTIS-CV [37] (Ertugliflozin)	3.5 yr	8,246	Age: 64.4 yr Men: 70.0%	BMI: 29.5 kg/m^2^ SBP: 133.4 mmHg LDLc: 2.3 mmol/L	HbA1c: 8.2% DM duration: 13.0 yr	HTN‡: 95.2% CVD: 100% HF: 23.7%	eGFR < 60: 21.9% ACR ≥ 30 mg/g: 39.3%		Statin 82.3% Antithrombotics 88.9% RAAS blockers 81.1%	3P-MACE 3.9/4.0 (100PY)
SCORED [8] (Sotagliflozin)	1.3 yr†	10,584	Age: 69 yr Men: 55.1%	BMI: 31.8 kg/m^2^ SBP: 138.5 mmHg	HbA1c: 8.3%	CVD: 48.6% HF: 31.0%	eGFR < 60: 100% ACR ≥ 30 mg/g: 65.0%		RAAS blockers 88.5%	3P-MACE 4.1/4.7 (100PY)
EXSCEL [28] (Exenatide)	3.3 yr†	14,752	Age: 62 yr Men: 62.0%	BMI: 32.7 kg/m^2^ SBP: 135.5 mmHg LDLc: 2.3 mmol/L	HbA1c: 8.1% DM duration: 13.1 yr	HTN‡: 90.3% CVD: 73.1% HF: 16.2%	eGFR < 60: 21.6% ACR ≥ 30 mg/g: 15.9%		Statin 73.5% Antithrombotics 73.4% RAAS blockers 85.0%	3P-MACE 3.7/4.0 (100PY)
LEADER [21] (Liraglutide)	3.8 yr†	9,340	Age: 64.3 yr Men: 64.3%	BMI: 32.5 kg/m^2^ SBP: 135.9 mmHg LDLc: 2.3 mmol/L	HbA1c: 8.7% DM duration: 12.7 yr	HTN: 90.0% CVD: 81.3% HF: 17.8%	eGFR < 60: 23.1% ACR ≥ 30 mg/g: 36.6%		Statin 72.2% Aspirin 69.8% RAAS blockers 77.2%	3P-MACE 3.4/3.9 (100PY)
REWIND [13] (Dulaglutide)	5.5 yr†	9,901	Age: 66.2 yr Men: 53.7%	BMI: 32.3 kg/m^2^ SBP: 137.2 mmHg LDLc: 2.6 mmol/L	HbA1c: 7.3% DM duration: 10.5 yr	HTN: 93.2% CVD: 31.5% HF: 8.6%	eGFR < 60: 22.2% ACR ≥ 30 mg/g: 35.0%		Statin 66.1% Antithrombotics 54.0% RAAS blockers 81.5%	3P-MACE 2.4/2.7 (100PY)
Harmony [19] (Albiglutide)	1.6 yr†	9,463	Age: 64.2 yr Men: 69.4%	BMI: 32.3 kg/m^2^ SBP: 134.7 mmHg LDLc: 2.1 mmol/L	HbA1c: 8.7% DM duration: 14.2 yr	HTN: 86.5% CVD: 100% HF: 20.3%	eGFR < 60: 23.5%		Statin 84.0% Antithrombotics 84.1% RAAS blockers 81.6%	3P-MACE 4.6/5.9 (100PY)
SUSTAIN-6 [29] (Semaglutide)	2.1 yr	3,297	Age: 64.6 yr Men: 60.7%	BMI: 32.8 kg/m^2^ SBP: 135.6 mmHg LDLc: 2.1 mmol/L	HbA1c: 8.7% DM duration: 13.9 yr	HTN: 92.8% CVD: 83.0% HF: 23.6%	eGFR < 60: 28.5% ACR ≥ 30 mg/g: 42.0%		Statin 72.8% Antithrombotics 76.3% RAAS blockers 83.5%	3P-MACE 3.2/4.4 (100PY)
PIONEER 6 [20] (Oral semaglutide)	1.0 yr†	3,183	Age: 66 yr Men: 68.4%	BMI: 32.3 kg/m^2^ SBP: 135.6 mmHg LDLc: 2.2 mmol/L	HbA1c: 8.2% DM duration: 14.9 yr	HTN‡: 95.3% CVD: 84.7% HF: 12.2%	eGFR < 60: 26.9%		Lipid-lowering agents 85.2% Antithrombotics 79.4%	3P-MACE 2.6/3.7 (100PY)
AMPLITUDE-O [18] (Efpeglenatide)	1.8 yr†	4,076	Age: 64.5 yr Men: 67.0%	BMI: 32.7 kg/m^2^ SBP: 134.9 mmHg LDLc: 2.1 mmol/L	HbA1c: 8.9% DM duration: 15.4 yr	HTN: 91.3% CVD: 89.5% HF: 18.1%	eGFR < 60: 31.6% ACR ≥ 30 mg/g: 48.5%		Statin 80.8% Aspirin 67.9% Other antiplatelets 25.7% RAAS blockers 80.0%	3P-MACE 5.4/6.8 (100PY)

BMI, body mass index; SBP, systolic blood pressure; LDLc, low-density lipoprotein cholesterol; HTN, hypertension; CVD, cardiovascular disease; HF, heart failure; eGFR, estimated glomerular filtration rate; ACR, albumin–creatinine ratio; RAAS, renin–angiotensin–aldosterone system; 3P-MACE, 3-point major adverse cardiovascular events; 100PY, 100 patient-year. * 3P-MACE event rate (100 patients-year) of active/placebo group. † Median and interquartile range converted to the mean value. ‡ Medically treated hypertension

### Overall 3P-MACE and risk of Bias

**Fig. 1:**
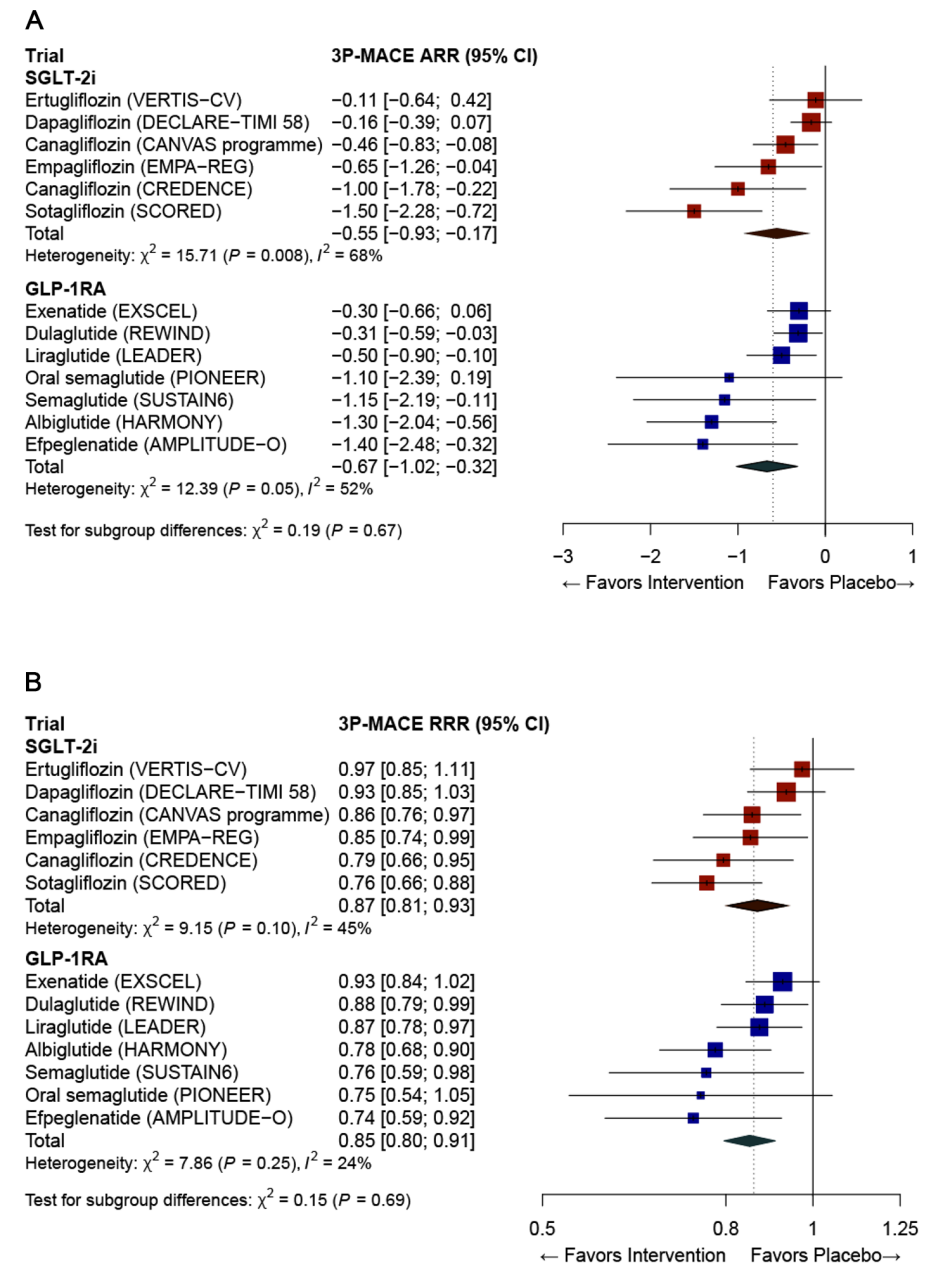
Absolute and relative risk reduction (ARR and RRR) in the incidence of a 3-point major adverse cardiovascular event (3P-MACE) in cardiovascular outcome trials with SGLT-2 inhibitor (SGLT-2i) or GLP-1 receptor agonist (GLP-1RA). The diamond indicates the pooled estimates, and the boxes are each study with 95% confidence interval. (**A**) Absolute risk reduction in the incidence of 3P-MACE by SGLT-2i and GLP-1RA. (**B**) Relative risk reduction in the incidence of 3P-MACE by SGLT-2i and GLP-1RA

The ARR and RRR of 3P-MACE calculated from six SGLT-2i trials and seven GLP-1RA trials are shown in Fig. 1. For all trial participants, compared with placebo, the ARR for 3P-MACE with SGLT-2is was − 0.55 per 100 person-years of follow-up (95% CI: − 0.93, − 0.17), which was slightly less pronounced than the − 0.67 per 100 person-years of follow-up (95% CI: − 1.02, − 0.32) with GLP-1RAs. Similarly, the RRR for 3P-MACE was slightly less pronounced with SGLT-2i therapy (RRR 0.87, 95% CI: 0.81, 0.93) than that with GLP-1RA therapy (RRR 0.85, 95% CI: 0.80, 0.91). All included trials were found to have high quality with a low risk of bias when the Cochrane risk of bias tool was applied (Supplementary Figure S2).

### Effect of SGLT-2is or GLP-1RAs on 3P-MACE by baseline eGFR or Albuminuria Status

**Fig. 2:**
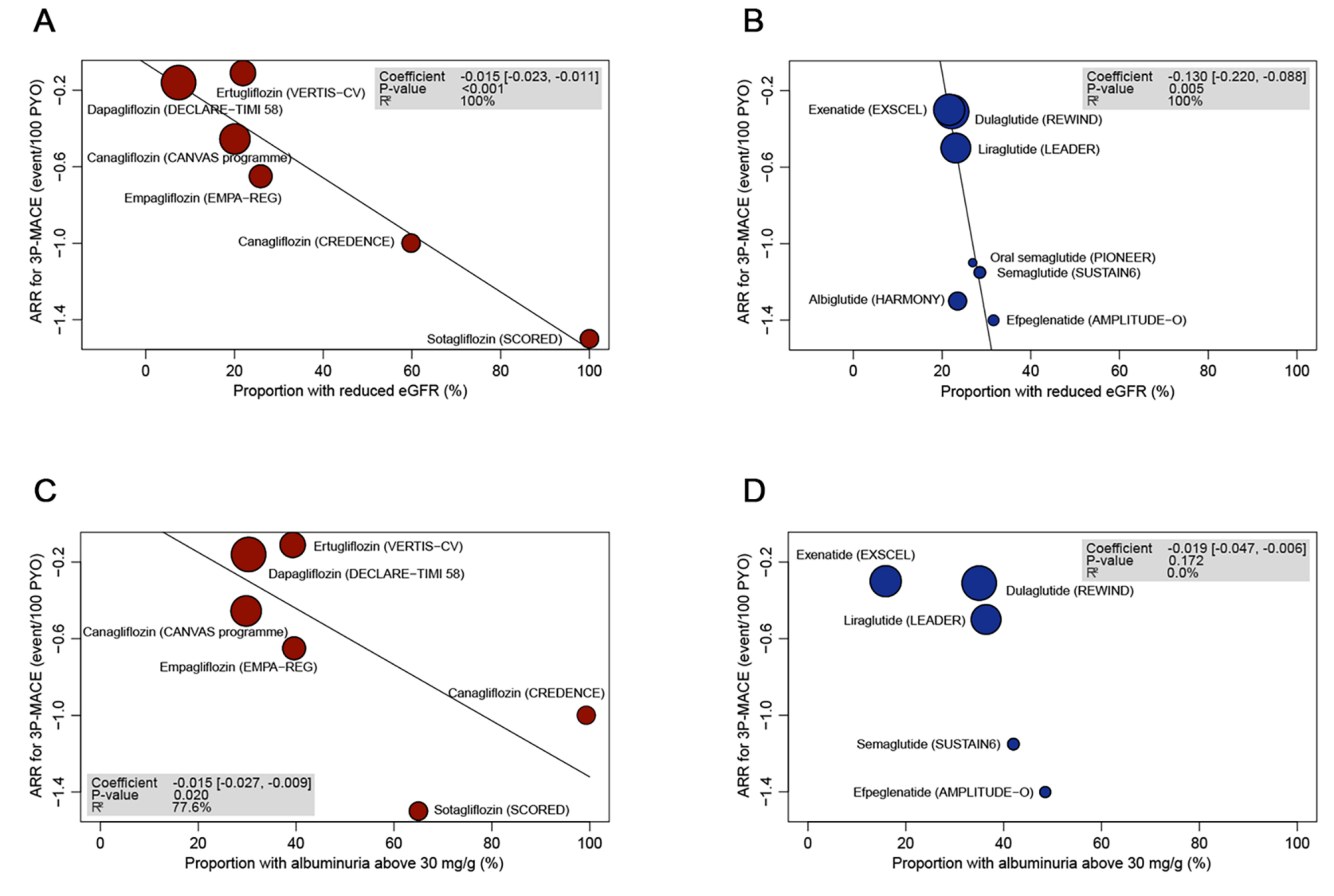
Meta-regression between ARR for 3P-MACE by SGLT-2i or GLP-1RA therapy and the proportion of patients with reduced eGFR (< 60 mL/min/1.73 m^2^) (A, B) or the proportion of patients with albuminuria (≥ 30 mg/g) (C, D). The coefficient represents the slope of the regression line, which is present when there is significance with P-value under 0.05. R^2^ indicates the strength of the association of the characteristics. ARR, absolute risk reduction; eGFR, estimated glomerular filtration ratio; GLP-1RA, glucagon-like peptide 1 receptor agonists; PYO, person-years of observation; SGLT-2i, sodium-glucose cotransporter-2 inhibitors; 3P-MACE, 3-point major adverse cardiovascular events. (**A**) Meta-regression between ARR for 3P-MACE by SGLT-2i therapy and the proportion of patients with reduced eGFR (< 60 mL/min/1.73 m^2^). (**B**) Meta-regression between ARR for 3P-MACE by GLP-1RA therapy and the proportion of patients with reduced eGFR (< 60 mL/min/1.73 m^2^). (**C**) Meta-regression between ARR for 3P-MACE by SGLT-2i therapy and proportion of patients with albuminuria (≥ 30 mg/g). (**D**) Meta-regression between ARR for 3P-MACE by GLP-1RA therapy and proportion of patients with albuminuria (≥ 30 mg/g)

**Fig. 3:**
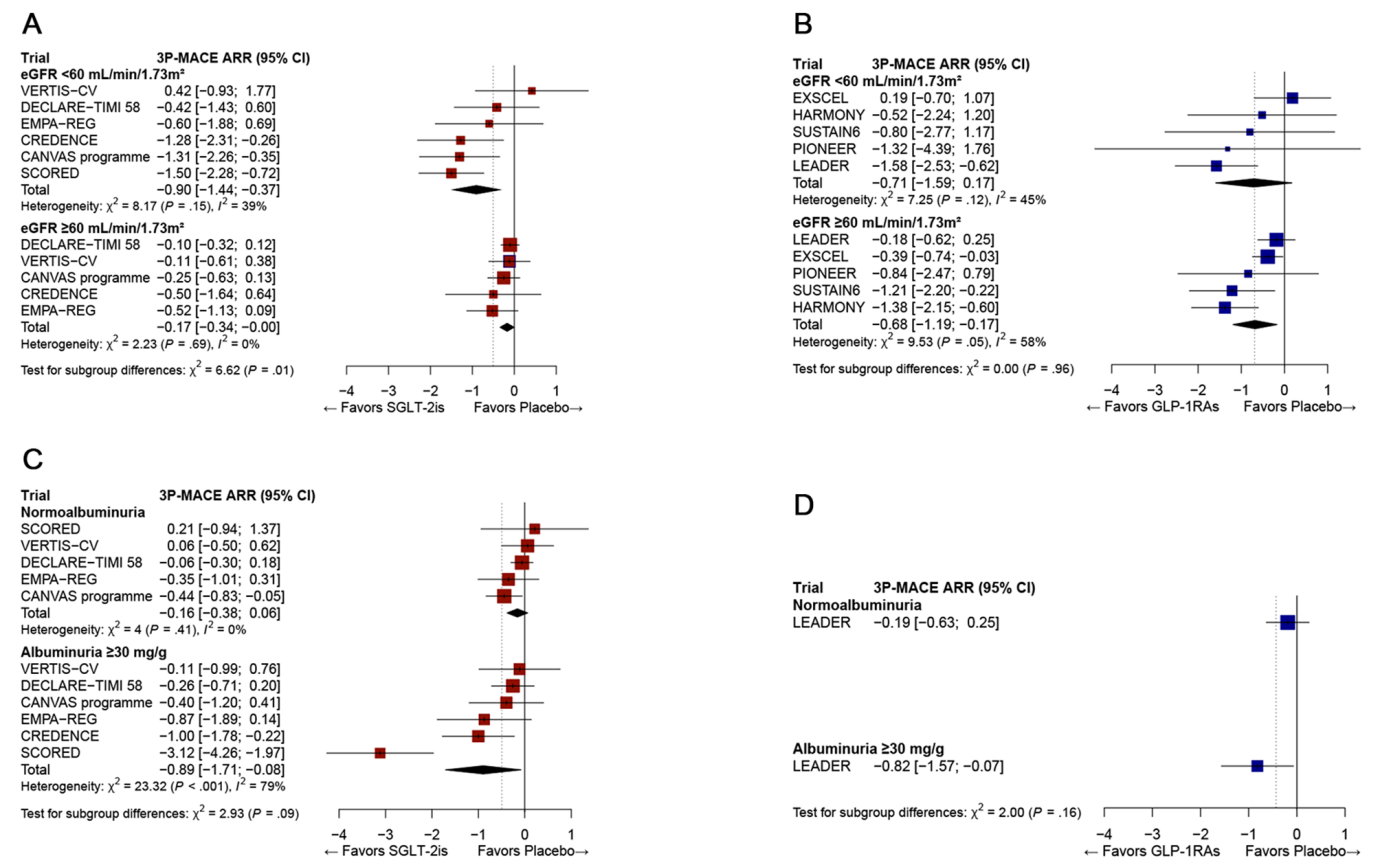
Comparison of absolute risk reduction for 3P-MACE according to baseline eGFR category and albuminuria status in SGLT-2i (A, C) or GLP-1RA (B, D) trials. The diamond indicates the pooled estimates, and the boxes are each study with 95% confidence interval. ARR, absolute risk reduction; eGFR, estimated glomerular filtration ratio; GLP-1RA, glucagon-like peptide 1 receptor agonists; SGLT-2i, sodium-glucose cotransporter-2 inhibitors; 3P-MACE, 3-point major adverse cardiovascular events. (**A**) Efficacy comparison on ARR for 3P-MACE according to baseline eGFR category in SGLT-2i trials. (**B**) Efficacy comparison on ARR for 3P-MACE according to baseline eGFR category in GLP-1RA trials. (**C**) Efficacy comparison on ARR for 3P-MACE according to albuminuria status in SGLT-2i trials. (**D**) Efficacy comparison on ARR for 3P-MACE according to albuminuria status in GLP-1RA trials

**Fig. 4:**
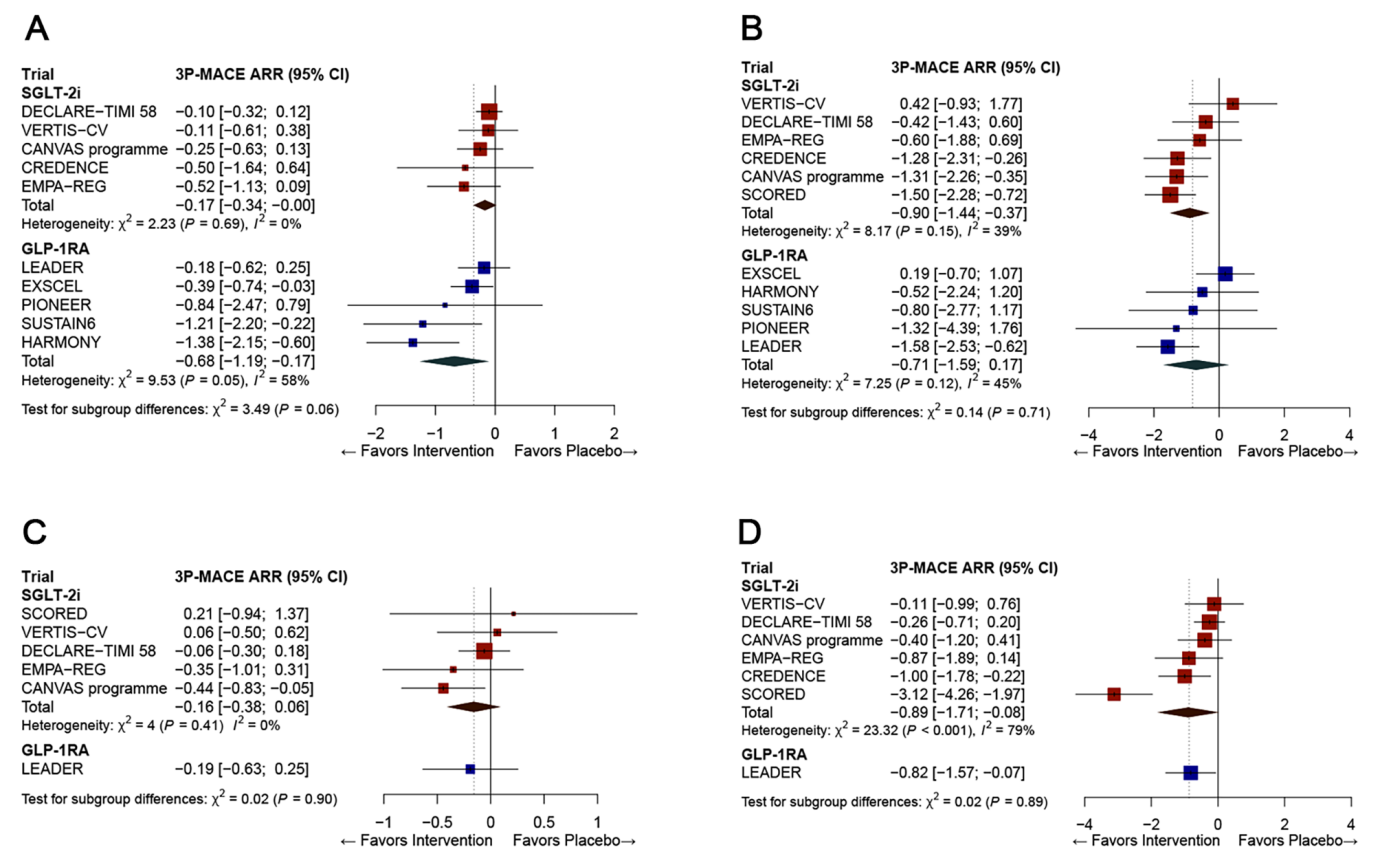
Efficacy comparison between SGLT-2i and GLP-1RA therapies on absolute risk reduction for 3P-MACE according to baseline eGFR category (A, B) and albuminuria status (C, D). The diamond indicates the pooled estimates, and the boxes are each study with 95% confidence interval. ARR, absolute risk reduction; eGFR, estimated glomerular filtration ratio; GLP-1RA, glucagon-like peptide 1 receptor agonists; SGLT-2i, sodium-glucose cotransporter-2 inhibitors; 3P-MACE, 3-point major adverse cardiovascular events. (**A**) Comparison between SGLT-2i and GLP-1RA therapy on ARR for 3P-MACE in normal eGFR. (**B**) Comparison between SGLT-2i and GLP-1RA therapy on ARR for 3P-MACE in reduced eGFR. (**C**) Comparison between SGLT-2i and GLP-1RA therapy on ARR for 3P-MACE in normoalbuminuria. (**D**) Comparison between SGLT-2i and GLP-1RA therapy on ARR for 3P-MACE in albuminuria

We investigated whether the effects of SGLT-2is or GLP-1RAs on 3P-MACE reduction would differ according to baseline renal function estimated by eGFR or albuminuria. For this, we conducted the meta-regression and meta-analyses with subgroups divided by the therapies (Figs. 2, 3 and 4 and Supplementary Figures S3–S5). All six SGLT-2i trials were included, but two GLP-1RA trials (REWIND [17] and AMPLITUDE-O [18]) were excluded because they did not report the 3P-MACE rate according to the baseline eGFR or albuminuria status.

The meta-regression analyses revealed significant negative associations, with strong strength (*R*^*2*^ = 100%), between the 3P-MACE incidence rates with SGLT-2i or GLP-1RA vs. placebo treatment and the proportions of the patients who had eGFR < 60 ml/min/1.73 m^2^ at baseline (Fig. 2 for ARR and Supplementary Figure S3 for RRR). This result suggests more favorable effects of two agents in people with reduced eGFR.

Notably, most study participants in GLP-1RA trials had a relatively good renal function: the participants with a baseline eGFR < 60 ml/min/1.73 m^2^ were fewer than 35%. The coefficient slopes relating the ARR in 3P-MACE to the proportion with eGFR < 60 ml/min/1.73 m^2^ were significantly steeper in the GLP-1RA studies than in the SGLT-2i studies.

Similarly, in the meta-analysis by eGFR subgroups, SGLT-2i therapy was more effective in reducing 3P-MACE in people with eGFR < 60 ml/min/1.73 m^2^, compared with those with ≥ 60 ml/min/1.73 m^2^ (ARR − 0.90, 95% CI: − 1.44, − 0.37 vs. ARR − 0.17, 95% CI: − 0.34, − 0.00; P = 0.01 for between-subgroup differences; Table 2). There was a trend for a greater effect of GLP-1RA therapy on 3P-MACE than that of SGLT-2i therapy in patients with normal eGFR: ARR − 0.68 (95% CI: − 1.19, − 0.17) vs. ARR − 0.17 (95% CI: − 0.34, − 0.00; P = 0.06; Fig. 4).

**Table 2:** Comparisons of Reductions in Absolute and Relative Risk for 3P-MACE Incidence with SGLT-2i or GLP-1RA Therapy According to Patient Characteristics

Subgroups	SGLT-2is					GLP-1RAs					
	Mean event rate in active / placebo groups (100 PYO)	Pooled ARR (95% CI)	*P*	Pooled RRR (95% CI)	*P*	Mean event rate in active / placebo groups (100 PYO)	Pooled ARR (95% CI)	*P*	Pooled RRR (95% CI)	*P*	
Overall	3.5 / 4.2	–0.55 (–0.93, − 0.17)		0.87 (0.81, 0.93)		3.6 / 4.4	–0.67 (–1.02, − 0.32)		0.85 (0.80, 0.91)		
eGFR ≥60 mL/min/1.73 m^2^	2.8 / 3.1	–0.17 (–0.34, − 0.01)	0.01	0.93 (0.86, 0.99)	0.09	3.2 / 4.0	–0.68 (–1.19, − 0.17)	0.96	0.83 (0.74, 0.94)	0.64	
eGFR < 60 mL/min/1.73 m^2^	4.4 / 5.2	–0.90 (–1.44, − 0.37)		0.83 (0.74, 0.92)		5.1 / 5.9	–0.71 (–1.59, 0.17)		0.88 (0.74, 1.04)		
Normoalbuminuria	2.9 / 3.0	–0.16 (–0.38, 0.06)	0.09	0.94 (0.87, 1.02)	0.13	2.8 / 3.0	–0.19 (–0.63, 0.25)	0.16	0.94 (0.80, 1.09)	0.34	
Albuminuria ≥30 mg/g	4.4 / 5.3	–0.89 (–1.71, − 0.08)		0.84 (0.75, 0.94)		4.3 / 5.2	–0.82 (–1.57, − 0.07)		0.82 (0.72, 0.99)		
Age < 65 years	2.7 / 3.0	–0.16 (–0.37, 0.05)	0.17	0.92 (0.85, 1.01)	0.41	3.0 / 4.0	–0.73 (–1.32, − 0.13)	0.59	0.82 (0.70, 0.95)	0.52	
Age ≥65 years	3.8 / 4.4	–0.54 (–1.04, − 0.05)		0.87 (0.78, 0.97)		4.0 / 4.7	–0.55 (–0.81, − 0.28)		0.86 (0.81, 0.92)		
Men	3.5 / 4.0	–0.36 (–0.65, − 0.08)	0.44	0.90 (0.83, 0.97)	0.91	4.0 / 4.8	–0.50 (–0.77, − 0.22)	0.61	0.88 (0.83, 0.94)	0.40	
Women	2.7 / 3.0	–0.22 (–0.45, 0.02)		0.89 (0.80, 0.99)		2.7 / 3.4	–0.40 (–0.65, − 0.16)		0.84 (0.76, 0.92)		
BMI < 30 kg/m^2^	3.1 / 3.6	–0.32 (–0.70, 0.06)	0.99	0.90 (0.81, 0.99)	0.99	3.4 / 4.6	–0.92 (–1.64, − 0.20)	0.30	0.80 (0.69, 0.94)	0.45	
BMI ≥30 kg/m^2^	3.3 / 3.7	–0.33 (–0.60, − 0.05)		0.90 (0.82, 0.98)		3.6 / 4.1	–0.52 (–0.74, − 0.30)		0.86 (0.80, 0.92)		
Low HbA1c	3.0 / 3.4	–0.31 (–0.63, 0.01)	0.90	0.90 (0.82, 0.99)	0.96	3.2 / 3.8	–0.31 (–0.56, − 0.06)	0.07	0.90 (0.83, 0.98)	0.16	
High HbA1c	3.4 / 3.9	–0.28 (–0.67, 0.11)		0.90 (0.82, 1.00)		3.8 / 4.8	–0.79 (–1.26, − 0.33)		0.83 (0.76, 0.90)		
No CVD	2.6 / 2.9	–0.20 (–0.75, 0.34)	0.41	0.91 (0.72, 1.16)	0.81	1.9 / 2.3	–0.21 (–0.73, 0.30)	0.12	0.89 (0.71, 1.12)	0.70	
Previous CVD	3.6 / 4.1	–0.44 (–0.67, − 0.21)		0.89 (0.83, 0.94)		4.1 / 5.0	–0.68 (–0.96, − 0.40)		0.85 (0.80, 0.91)		
No HF	2.8 / 3.3	–0.29 (–0.52, − 0.06)	0.66	0.89 (0.83, 0.96)	0.28	3.4 / 3.9	–0.42 (–0.67, − 0.17)	0.63	0.88 (0.82, 0.95)	0.97	
Previous HF	5.0 / 5.2	–0.14 (–0.77, 0.48)		0.97 (0.85, 1.10)		5.7 / 6.7	–0.81 (–2.38, 0.76)		0.88 (0.71, 1.10)		

*P* for subgroup differences. ARR, absolute risk reduction, RRR: relative risk reduction; 3P-MACE, 3-point major adverse cardiovascular events; SGLT-2i, sodium–glucose cotransporter-2 inhibitor; GLP-1RA, glucagon-like peptide-1 receptor agonist; PYO, person-years of observation; CI, confidence interval

In terms of albuminuria status, meta-regression analyses showed a negative association between the ARR for 3P-MACE and the proportions of people who had albuminuria ≥ 30 mg/g at baseline in SGLT-2i trials, with strong strength (*R*^*2*^ = 77.6%; Fig. 2). In GLP-1RA trials, HARMONY [19] and PIONEER-6 [20] trials were excluded because they did not provide results by albuminuria status (≥ 30 vs. < 30 mg/g).

Similar results were found in the meta-analysis (Fig. 3). SGLT-2i therapy effectively reduced 3P-MACE in people with albuminuria ≥ 30 mg/g at baseline (ARR − 0.89, 95% CI: − 1.71, − 0.08) but not in those with normoalbuminuria (ARR − 0.16, 95% CI: − 0.38, 0.06). However, there was no difference between subgroups (P = 0.09). For the GLP-1RA class, the LEADER trial alone [21] reported the subgroup results according to the baseline albuminuria status. In this study, liraglutide therapy effectively reduced 3P-MACE in people with albuminuria at baseline but not in those without albuminuria, without a significant between-subgroup difference (P = 0.16).

### Efficacy comparison between SGLT-2is and GLP-1RAs on 3P-MACE according to baseline eGFR category or Albuminuria Status

In the meta-analysis, GLP-1RA therapy was more effective in the ARR of 3P-MACE in patients with normal renal function than SGLT-2 therapy (Fig. 4). In contrast, the beneficial effects of SGLT-2i and GLP-1RA therapies on the ARR of 3P-MACE were not different in the patients with reduced renal function.

Concerning the albuminuria status, the beneficial effects of SGLT-2i and GLP-1RA therapies on the ARR of 3P-MACE did not differ according to this characteristic. However, only one study (LEADER study [21]) was included in the GLP-1RA class.

### 3P-MACE reduction with SGLT-2i and GLP-1RA by other baseline characteristics

The effects of SGLT-2i or GLP-1RA therapy on 3P-MACE by age, sex, BMI, baseline HbA1c level, preexisting CVD, and preexisting HF were also examined (Supplementary Figures S6–S11). Among the SGLT-2i studies, the SCORED trial [8] was not included in these subgroup meta-analyses because it did not report the results by these characteristics. All seven GLP-1RA trials were included in these subgroup meta-analyses. However, three GLP-1RA trials were included for the meta-analysis by preexisting HF.

Comparing subgroups defined by age, sex, BMI, HbA1c, and proportion with preexisting CVD or HF, both the ARR and RRR were not significantly different (Table 2; Supplementary Figures S6–S11). GLP-1RAs exhibit better efficacy for 3P-MACE reduction in patients with high HbA1c levels vs. those with low HbA1c levels without significance (Table 2). The ARR in 3P-MACE was greater with GLP-1RA therapy compared with SGLT-2i therapy in the patients under 65 years of age (–0.73, 95% CI: − 1.32, − 0.13 vs. − 0.16, 95% CI: − 0.37, 0.05 for 3P-MACE events per 100 person-years of observation; P = 0.08; Supplementary Figure S6C). This may suggest the better effect of GLP-1RA treatment at a young age.

### Quality Assessment

Supplementary table S1 presents the GRADE evidence profiles for the subgroups. Nine meta-analyses had inconsistency with moderate heterogeneity. Limited reporting of results by the status of previous CVD and HF history resulted in downgrades in certainty. As only the LEADER trial reported 3P-MACE by albuminuria status, the analyses were downgraded two levels. Finally, four meta-analyses were given low certainty: (1–2) subgroups divided by albuminuria in GLP-1RA trials; (3) patients without previous CVD in SGLT-2i trials; and (4) patients with HF history in GLP-1RA trials.

## Discussion

In CVOTs, SGLT-2i and GLP-1RA therapies were significantly and similarly effective in 3P-MACE reduction, providing benefits of 13% and 15% RRR and 0.55 and 0.67 ARR (event/100 person-years of observation), respectively. In the meta-regression of 13 randomized placebo-controlled trials examining SGLT-2i and GLP-1RA treatments, there was a negative association between poorer renal dysfunction (decreased eGFR) and greater ARR for 3P-MACE. The presence of albuminuria was also linked to a greater ARR with SGLT-2i therapy. In the meta-analysis by eGFR subgroups, SGLT-2i therapy was more effective in reducing 3P-MACE in people with eGFR < 60 ml/min/1.73 m^2^ compared with those with normal renal function (Fig. 3). At the same time, this difference was much less prominent with GLP-1RA therapy.

Both reduced eGFR and albuminuria are independently associated with a higher risk of cardiovascular events in people with T2DM [22]. Recent studies on people with established chronic renal failure have proven that SGLT-2i effectively reduces cardiovascular fatality and a composite renal outcome [6, 7]. Thus, in addition to the previously reported renal benefits of SGLT-2i therapy in patients with T2DM [23–25], our data support the use of SGLT-2i therapy in this subgroup at high risk for both cardiovascular and renal complications.

It is obvious that both SGLT-2i and GLP-1RA therapy help reduce 3P-MACE in patients with renal impairment, who generally exhibit a high risk for CVD [26]. Intriguingly, the ARR in people with normal renal function seemed to be larger with GLP-1RA therapy than with SGLT-2i therapy (–0.68 vs. − 0.17; P = 0.06; Fig. 3). However, it should be noted that GLP-1RA trials included a relatively narrow spectrum in the proportion of people with eGFR < 60 ml/min/1.73 m^2^ than SGLT-2i trials (21.6–31.6% vs. 7.4–100%, P < 0.05). Thus, it might have been difficult to identify the different effects of GLP-1RA on 3P-MACE reduction according to the eGFR level. On the contrary, the lack of benefit observed in GLP-1RA therapy in patients with low eGFR may have resulted from insufficient statistical power caused by the narrow range of eGFR in the study participants [27]. Taken together, our analysis data including the most available studies indicate that GLP-1RA therapy may be beneficial even for people with normal renal function, which is not the case for SGLT-2i therapy.

Liraglutide therapy in the LEADER trial reduced 3P-MACE significantly more in patients with eGFR < 60 ml/min/1.73 m^2^ than in those with ≥ 60 ml/min/1.73 m^2^ [21], while other GLP-1RAs such as exenatide, albiglutide, and semaglutide tended to reduce 3P-MACE more in patients with normal eGFR [19, 28, 29]. This finding suggests that individual GLP-1RAs might have varying effects on 3P-MACE reduction according to the baseline renal function, but data are not enough to draw a conclusion indicating a drug-specific efficacy of GLP-1RA rather than the class effect. More studies with participants with a wide spectrum of eGFR are needed.

Subjects with albuminuria at baseline showed a tendency of greater reduction in the 3P-MACE by both SGLT-2i and GLP-1RA therapies. Treatment with SGLT-2is is known to significantly reduce albuminuria [30, 31], and reduction in albuminuria in the first year was associated with long-term cardiovascular benefits [31]. Among SGLT-2i trials, sotagliflozin therapy in SCORED showed remarkable efficacy in 3P-MACE reduction in patients with albuminuria [8]. Since the SCORED trial included only patients with low eGFR, with 65.0% of them had albuminuria, it can be speculated that the protective effect of SGLT-2is could be more pronounced in such patients [32]. Only the LEADER trial reported the subgroup results by albuminuria, which hinders the gathering of important data regarding the potential influences of albuminuria on the cardioprotective effects of GLP-1RA class.

Regarding albuminuria, renin–angiotensin–aldosterone system (RAAS) blockers might be intertwined in response to SGLT-2i or GLP-1RA therapy. However, RAAS blockers were used in over 80% of all 13 trials without significant differences (Table 1). In addition, no associations between the use of RAAS blockers and the efficacy of SGLT-2i or GLP-1RAs on 3P-MACE were observed in the meta-regression analysis.

Other characteristics, including age, sex, BMI, HbA1c level at baseline, preexisting CVD, or preexisting HF status, did not significantly affect the effects of SGLT-2i and GLP-1RA therapies on the ARR or RRR of 3P-MACE. However, GLP-1RA therapy tended to be more effective in the ARR of 3P-MACE than SGLT-2i therapy, with borderline significance in the younger age group (Supplementary Figure S6). It is also noteworthy that, compared with the SGLT-2i therapy, GLP-1RA therapy tended to exhibit better efficacy for 3P-MACE reduction in people with uncontrolled diabetes vs. those with controlled diabetes (Table 2).

A previous study with a different approach reported that people with established CVD showed a greater reduction in 3P-MACE by GLP-1RA and SGLT-2i therapies compared with those with risk factors alone (difference in effect between patients with vs. without a history of CVD: P = 0.049) [33]. However, that study included CVOTs up to June 2019. In our study, which included the CVOTs up to 2022, CVD was associated with a greater reduction in 3P-MACE by SGLT-2i and GLP-1RA. There was no statistically significant difference in the between-group analysis. Taken together, it is obvious that more trials are needed to confirm such effects of patient characteristics. Still, our data do not support the differential use of SGLT-2is and GLP-1RAs with the expectation to improve the prognosis by individualizing treatment considering these baseline characteristics.

The present analysis could be improved in a number of ways. First, the relatively small number of studies is likely to reduce the power needed to find significance between subgroups, particularly in meta-regression analysis [34]. Thus, nonsignificant results cannot rule out the significant impact of certain characteristics on the efficacy of SGLT-2i or GLP-1RA therapy in 3P-MACE reduction. Second, studies that did not report the 3P-MACE results by subgroups were not included for the analysis, reducing the statistical power of the analysis. Some subgroups were limited in representation: e.g., studies with GLP-1RAs in patients with impaired renal function at baseline. Third, each compound was only tested against placebo, and there were no head-to-head comparison studies between SGLT-2is and GLP-1RAs regarding cardiovascular effects. Thus, all comparisons are indirect and may be confounded by differences in unidentified patients characteristics, such as concomitant medical therapy addressing hypertension, lipid abnormalities, and other conditions typically associated with T2DM. Nonetheless, we included all large CVOTs currently available, and we believe that the results obtained by meta-regression and meta-analysis provide meaningful information about the proper use of these two novel agents.

## Conclusions

In the meta-regression and meta-analyses, SGLT-2i therapy was more effective in the ARR of 3P-MACE in patients with decreased renal function (significant) or albuminuria (trend) than in those without. In contrast, the beneficial effects of GLP-1RA therapy on the ARR of 3P-MACE did not differ for these subgroups. However, the effect of GLP-1RA therapy was more pronounced in patients with high HbA1c levels at baseline than in those without.

Both classes showed a significant positive association between the efficacy of the reduction of 3P-MACE and the proportion of patients with reduced renal function. SGLT-2i therapy was effective in the 3P-MACE in both groups of patients with normal and reduced eGFR, with greater efficacy in reduced eGFR. In contrast, the efficacy of GLP-1RA therapy of 3P-MACE was similar in both groups, but significant only in those with normal eGFR. Our findings support the use of SGLT-2i in patients with impaired renal function and GLP-1RA in patients with normal renal function for optimizing the differential prescription to prevent major adverse cardiovascular events.

## Electronic supplementary material

Below is the link to the electronic supplementary material.

Supplementary Material 1

## Data Availability

Data are included in the tables and figures.

## Abbreviations

ARR: absolute risk reduction
ASCVD: Atherosclerotic cardiovascular disease
BMI: Body mass index
CKD: Chronic kidney disease
CI: Confidence interval
CVD: Cardiovascular death
CVOT: Cardiovascular outcome trials
DM: Diabetes mellitus
eGFR: Estimated glomerular filtration rate
ESRD: End-stage renal disease
GLP-1RA: Glucagon-like peptide-1 receptor agonist
HF: Heart failure
MACE: Major adverse cardiovascular events
PRISMA: Preferred reporting items for systematic reviews and meta-analyses
RCT: Randomized control trials
RR: Risk ratio
RRR: relative risk reduction
SGLT-2i: Sodium-glucose cotransporter-2 inhibitor
T2DM: type 2 diabetes mellitus
3P-MACE: 3-point major adverse cardiovascular events
