# Supplementary material for: Characteristics predicting the efficacy of SGLT-2 inhibitors versus GLP-1 receptor agonists on major adverse cardiovascular events in type 2 diabetes mellitus: a meta-analysis study
Source: Cardiovasc Diabetol. 2023 Jun 28;22:153. doi: 10.1186/s12933-023-01877-6 (PMC10303335; doi:10.1186/s12933-023-01877-6)
Supplement: Supplementary file 1 — Supplementary Material 1 [file 12933_2023_1877_MOESM1_ESM.docx]

**Supplementary Figure S1. Flow diagram for study selection.**

**
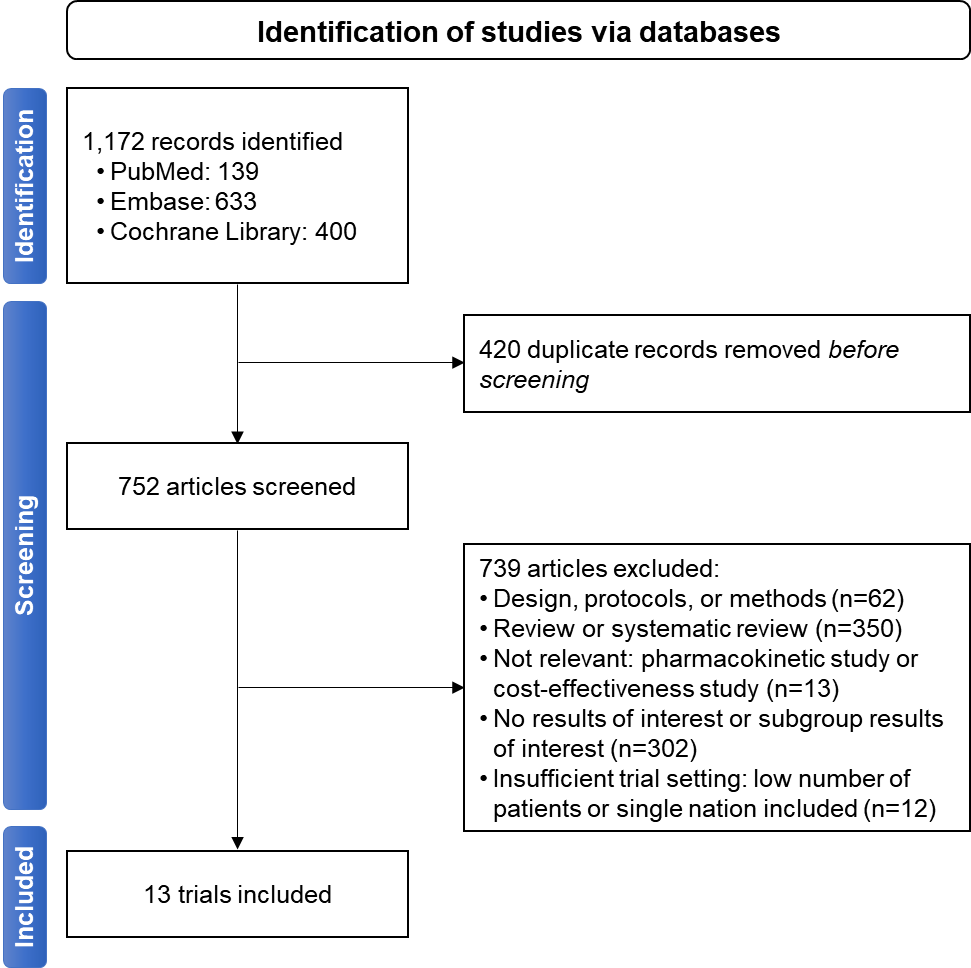
**

**Supplementary Figure S2. Risk of bias among trials included in the meta-regression.**

in green color indicates a low risk of bias.

| CANVAS programme | CREDENCE | DECLARE-TIMI 58 | EMPA-REG OUTCOME | VERTIS-CV | SCORED | EXSCEL | LEADER | REWIND | Harmony | SUSTAIN-6 | PIONEER 6 | AMPLITUDE-O |  |
| --- | --- | --- | --- | --- | --- | --- | --- | --- | --- | --- | --- | --- | --- |
|  |  |  |  |  |  |  |  |  |  |  |  |  | Bias arising from the randomization process |
|  |  |  |  |  |  |  |  |  |  |  |  |  | Bias due to deviations from intended interventions |
|  |  |  |  |  |  |  |  |  |  |  |  |  | Bias due to missing outcome data |
|  |  |  |  |  |  |  |  |  |  |  |  |  | Bias in measurement of the outcome |
|  |  |  |  |  |  |  |  |  |  |  |  |  | Bias in selection of the reported result |

**Supplementary Figure S3. Meta-regression between RRR for 3P-MACE by SGLT-2i or GLP-1RA therapy and the proportion of patients with reduced eGFR (< 60 mL/min/1.73 m^2^) (A, B) or the proportion of patients with albuminuria (**≥ **30 mg/g) (C, D).** The coefficient represents the slope of the regression line, which is present when there is significance with P-value under 0.05. R^2^ indicates the strength of the association of the characteristics. eGFR, estimated glomerular filtration ratio; GLP-1RA, glucagon-like peptide 1 receptor agonists; PYO, person-years of observation; RRR, relative risk reduction; SGLT-2i, sodium-glucose cotransporter-2 inhibitors; 3P-MACE, 3-point major adverse cardiovascular events.

| (A) Meta-regression between RRR for 3P-MACE by SGLT-2i therapy and the proportion of patients with reduced eGFR (< 60 mL/min/1.73 m^2^) | (B) Meta-regression between RRR for 3P-MACE by GLP-1RA therapy and the proportion of patients with reduced eGFR (< 60 mL/min/1.73 m^2^) |
| --- | --- |
| 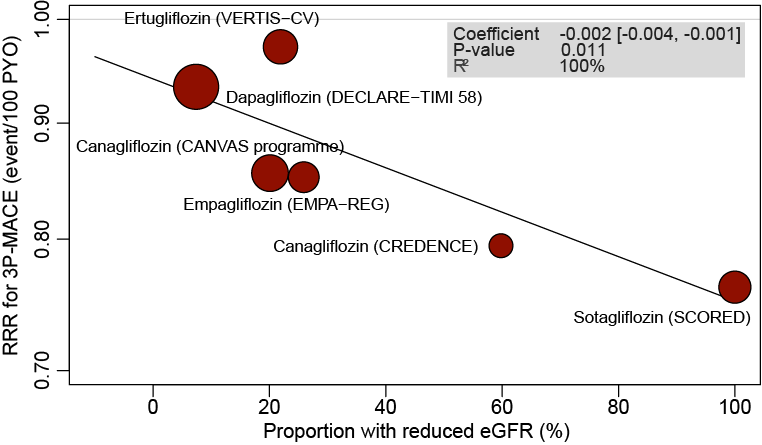 | 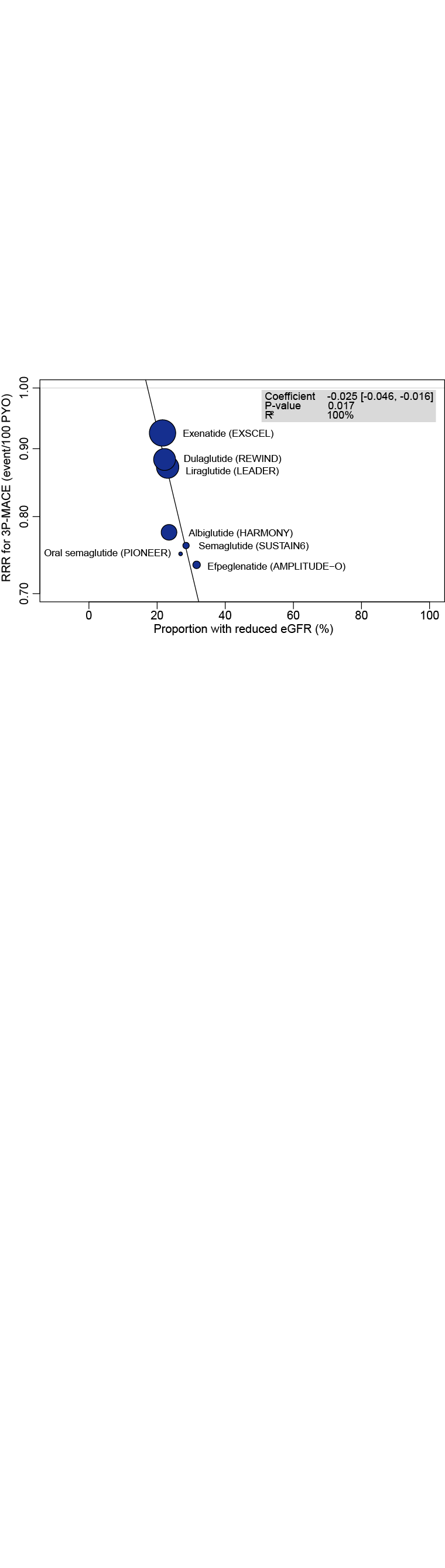 |
| (C) Meta-regression between RRR for 3P-MACE by SGLT-2i therapy and proportion of patients with albuminuria (≥ 30 mg/g) | (D) Meta-regression between RRR for 3P-MACE by GLP-1RA therapy and proportion of patients with albuminuria (≥ 30 mg/g) |
| 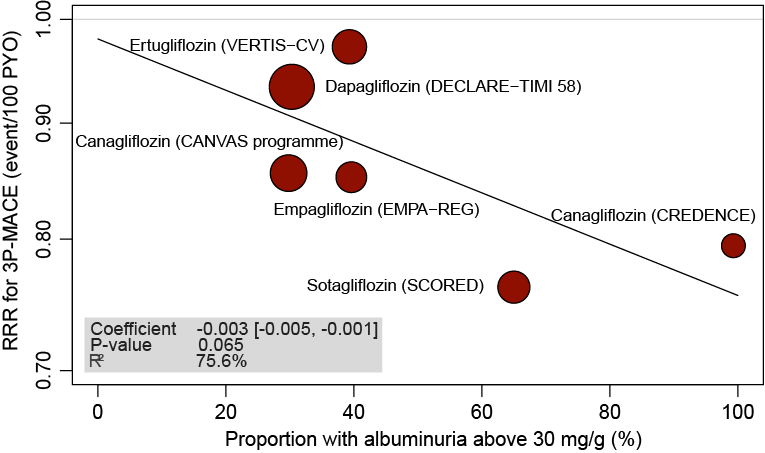 | 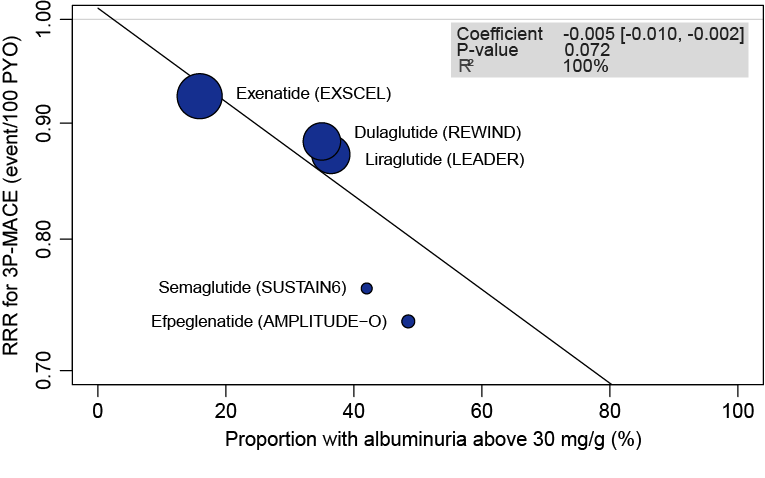 |

**Supplementary Figure S4. Comparison of RRR for 3P-MACE according to baseline eGFR category and albuminuria status in SGLT-2i (A, C) or GLP-1RA (B, D) trials.** The diamond indicates the pooled estimates, and the boxes are each study with 95% CI. CI, confidence interval; eGFR, estimated glomerular filtration ratio; GLP-1RA, glucagon-like peptide 1 receptor agonists; RRR, relative risk reduction; SGLT-2i, sodium-glucose cotransporter-2 inhibitors; 3P-MACE, 3-point major adverse cardiovascular events.

| (A) Efficacy comparison on RRR for 3P-MACE according to baseline eGFR category in SGLT-2i trials | (B) Efficacy comparison on RRR for 3P-MACE according to baseline eGFR category in GLP-1RA trials |
| --- | --- |
| 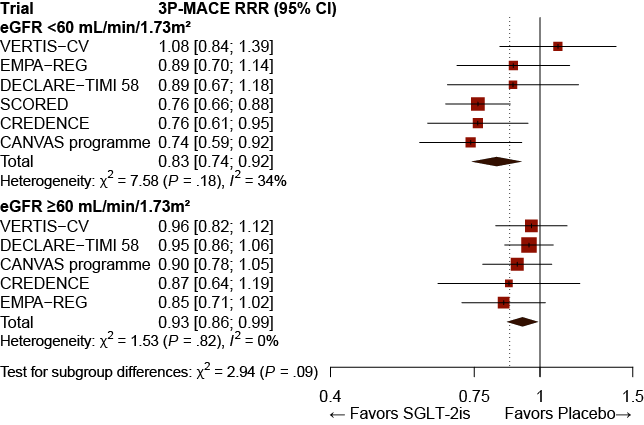 | 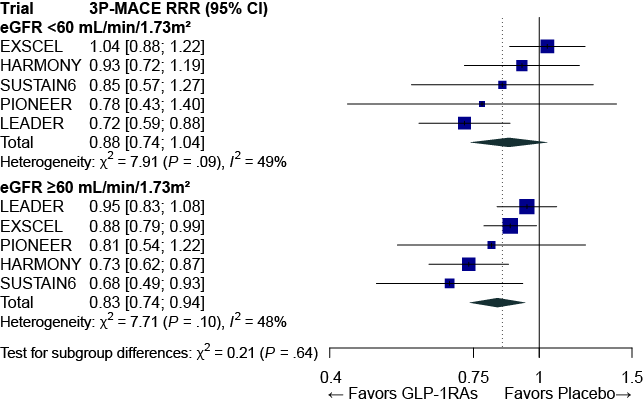 |
| (C) Efficacy comparison on RRR for 3P-MACE according to albuminuria status in SGLT-2i trials | (D) Efficacy comparison on RRR for 3P-MACE according to albuminuria status in GLP-1RA trials |
| 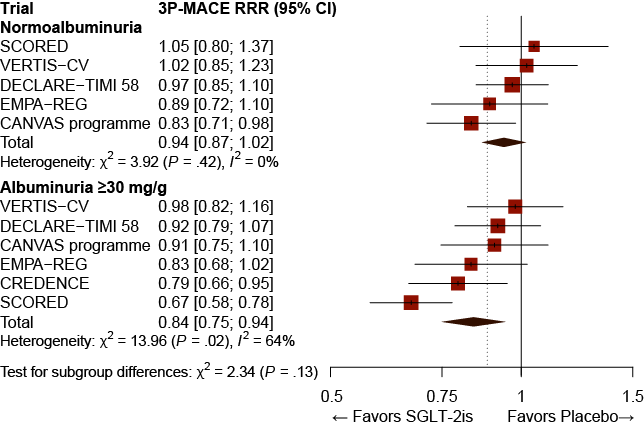 | 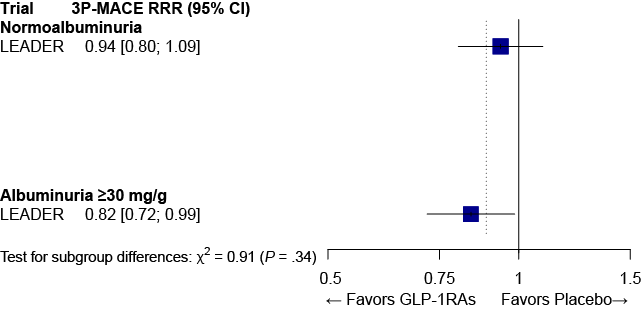 |

**Supplementary Figure S5. Efficacy comparison between SGLT-2i and GLP-1RA therapies on relative risk reduction for 3P-MACE according to baseline eGFR category (A, B) and albuminuria status (C, D).** The diamond indicates the pooled estimates, and the boxes are each study with 95% CI. CI, confidence interval; eGFR, estimated glomerular filtration ratio; GLP-1RA, glucagon-like peptide 1 receptor agonists; PYO, person-years of observation; RRR, relative risk reduction; SGLT-2i, sodium-glucose cotransporter-2 inhibitors; 3P-MACE, 3-point major adverse cardiovascular events.

| (A) Comparison between SGLT-2i and GLP-1RA therapy on RRR for 3P-MACE in normal eGFR | (B) Comparison between SGLT-2i and GLP-1RA therapy on RRR for 3P-MACE in reduced eGFR |
| --- | --- |
| 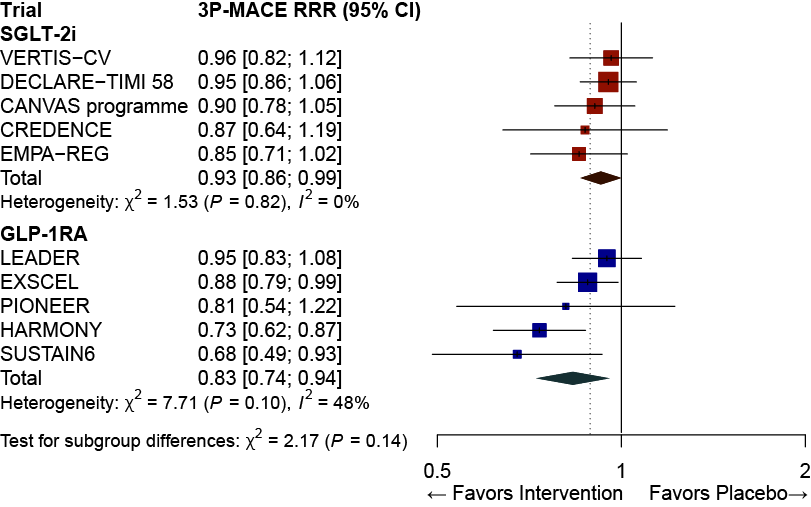 | 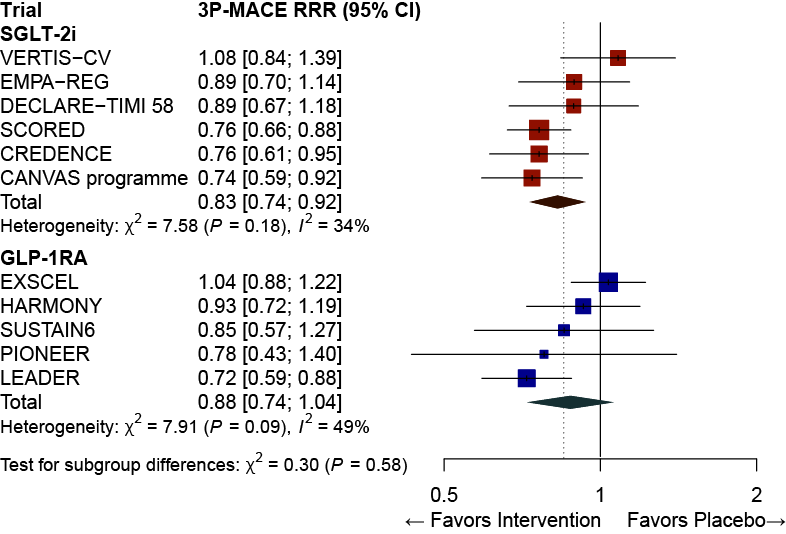 |
| (C) Comparison between SGLT-2i and GLP-1RA therapy on RRR for 3P-MACE in normoalbuminuria | (D) Comparison between SGLT-2i and GLP-1RA therapy on RRR for 3P-MACE in albuminuria |
| 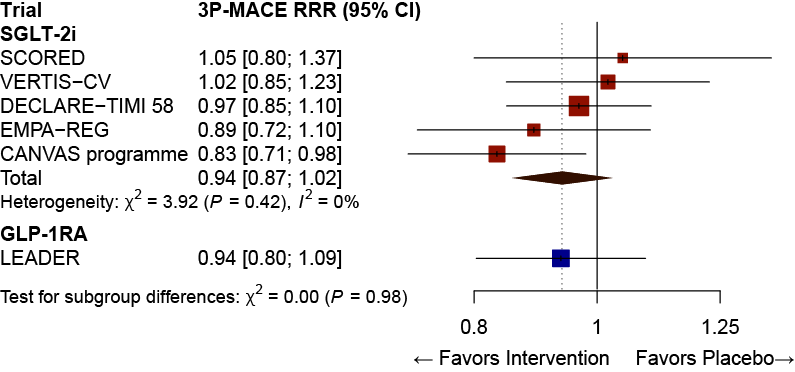 | 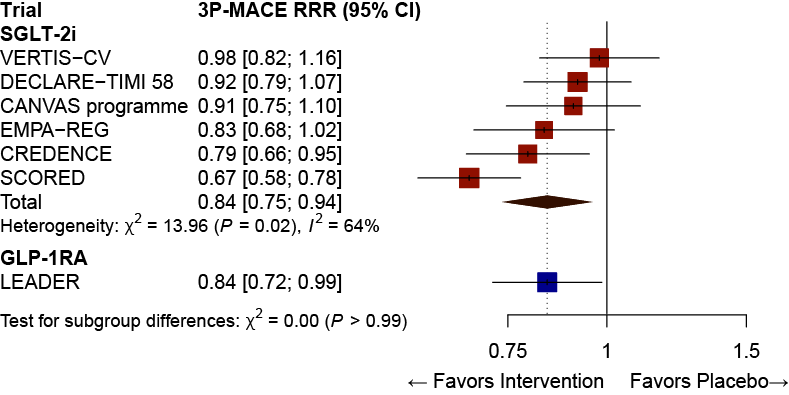 |

**Supplementary Figure S6. Absolute risk reduction (A~D) and Relative risk reduction (E~H) for 3P-MACE by baseline age.** The coefficient represents the slope of the regression line. R^2^ indicates the strength of the association of the characteristics. The diamond indicates the pooled estimates, and the boxes are each study with 95% CI. ARR, absolute risk reduction; CI, confidence interval; GLP-1RA, glucagon-like peptide 1 receptor agonists; PYO, person-years of observation; RRR, relative risk reduction; SGLT-2i, sodium-glucose cotransporter-2 inhibitors; 3P-MACE, 3-point major adverse cardiovascular events.

| **(A) Meta-regression between ARR for 3P-MACE by SGLT-2i therapy and average age** | **(B) Meta-regression between ARR for 3P-MACE by GLP-1RA therapy and average age** |
| --- | --- |
| 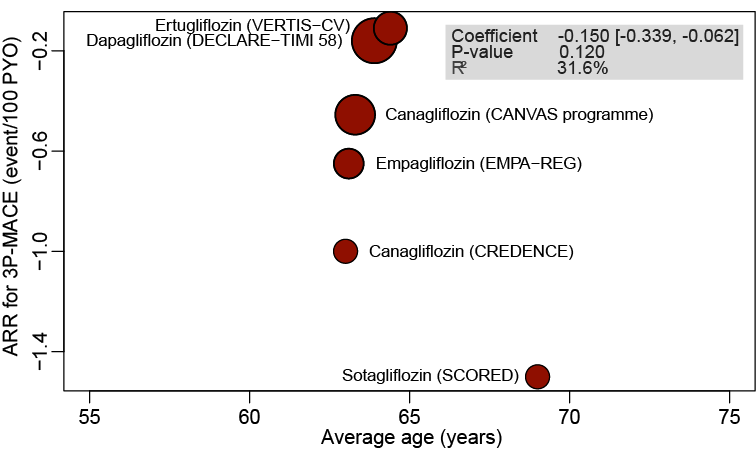 | 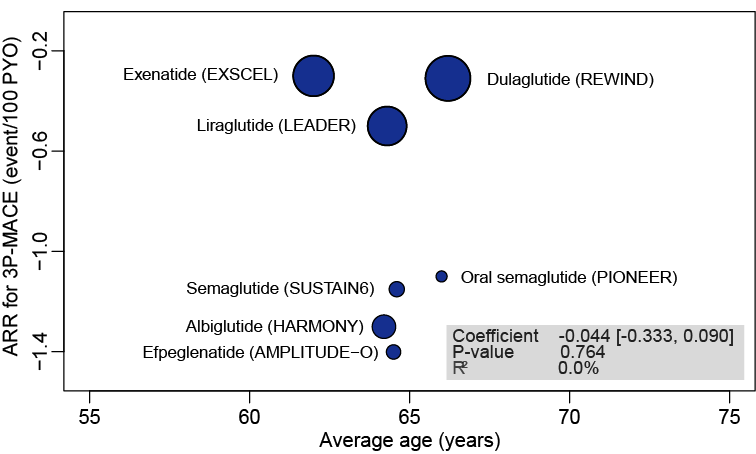 |
| **(C) Efficacy of SGLT-2i and GLP-1RA therapy on ARR for 3P-MACE in younger age group** | **(D) Efficacy of SGLT-2i and GLP-1RA therapy on ARR for 3P-MACE in older age group** |
| 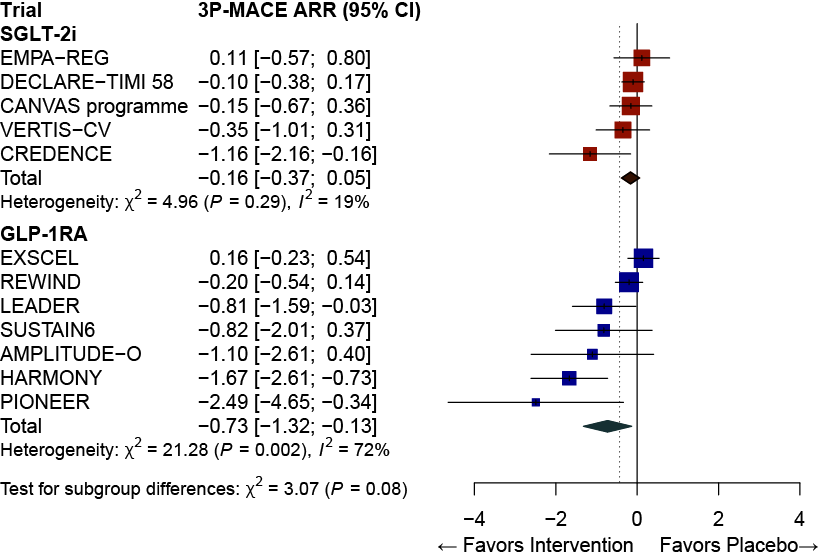 | 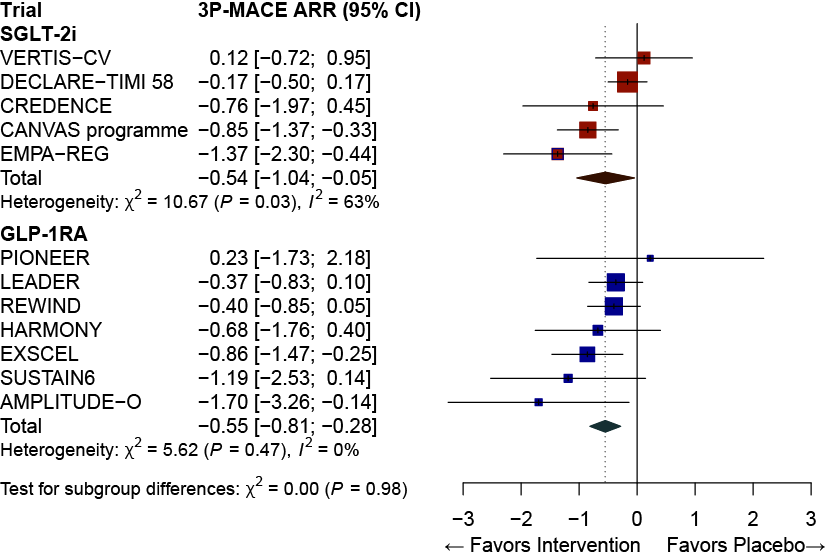 |

| **(E) Meta-regression between RRR for 3P-MACE by SGLT-2i therapy and average age** | **(F) Meta-regression between RRR for 3P-MACE by GLP-1RA therapy and average age** |
| --- | --- |
| 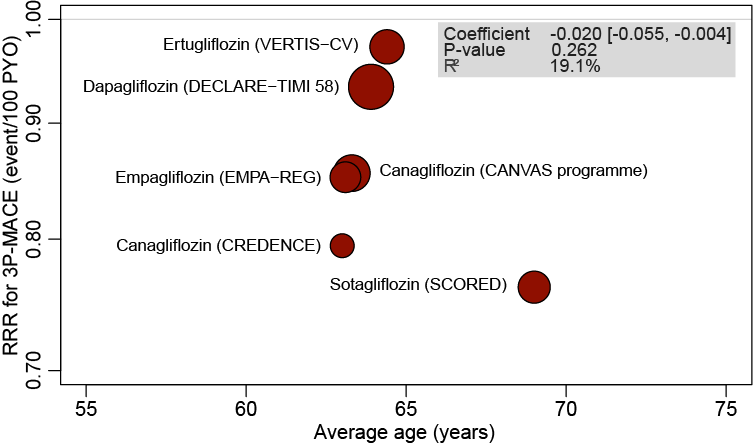 | 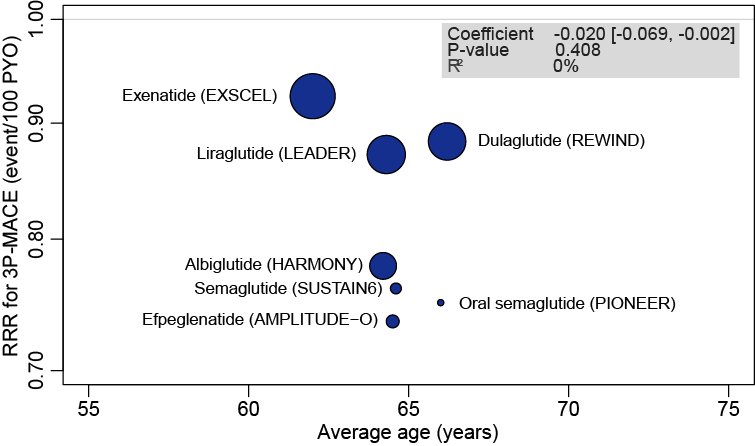 |
| **(G) Efficacy of SGLT-2i and GLP-1RA therapy on RRR for 3P-MACE in younger age group** | **(H) Efficacy of SGLT-2i and GLP-1RA therapy on RRR for 3P-MACE in older age group** |
| 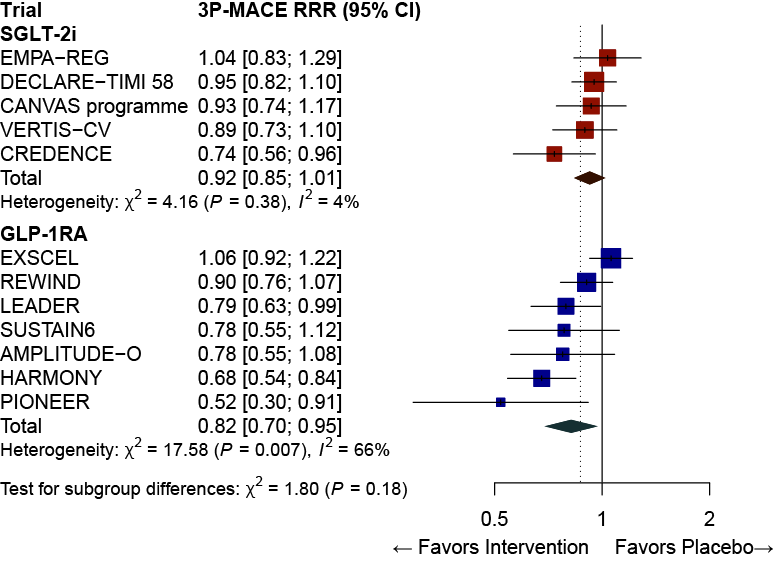 | 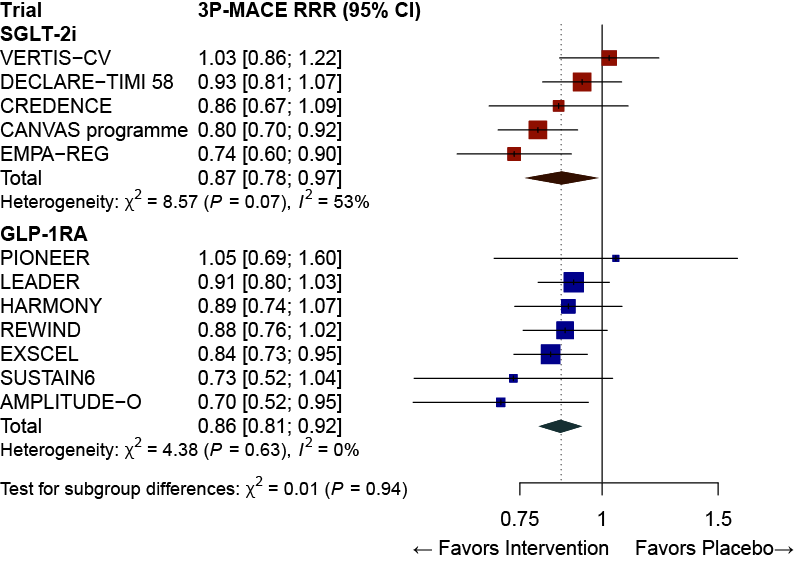 |

**Supplementary Figure S7. Absolute risk reduction (A~D) and Relative risk reduction (E~H) for 3P-MACE by baseline sex.** The coefficient represents the slope of the regression line. R^2^ indicates the strength of the association of the characteristics. The diamond indicates the pooled estimates, and the boxes are each study with 95% CI. ARR, absolute risk reduction; CI, confidence interval; GLP-1RA, glucagon-like peptide 1 receptor agonists; PYO, person-years of observation; RRR, relative risk reduction; SGLT-2i, sodium-glucose cotransporter-2 inhibitors; 3P-MACE, 3-point major adverse cardiovascular events.

| **(A) Meta-regression between ARR for 3P-MACE by SGLT-2i therapy and the proportion of men** | **(B) Meta-regression between ARR for 3P-MACE by GLP-1RA therapy and the proportion of men** |
| --- | --- |
| 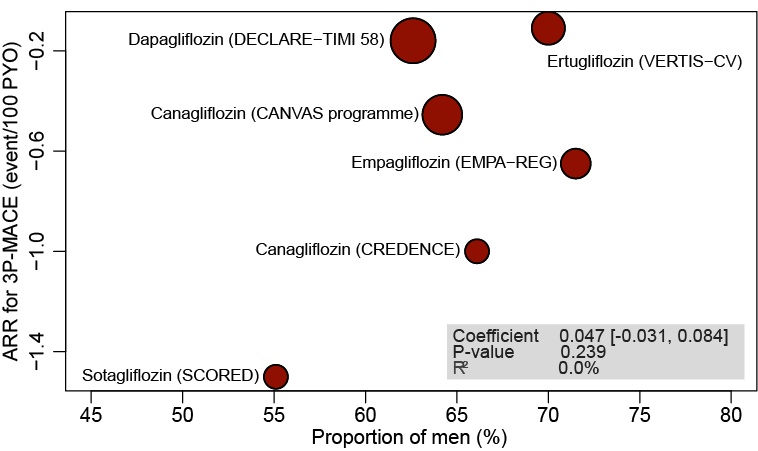 | 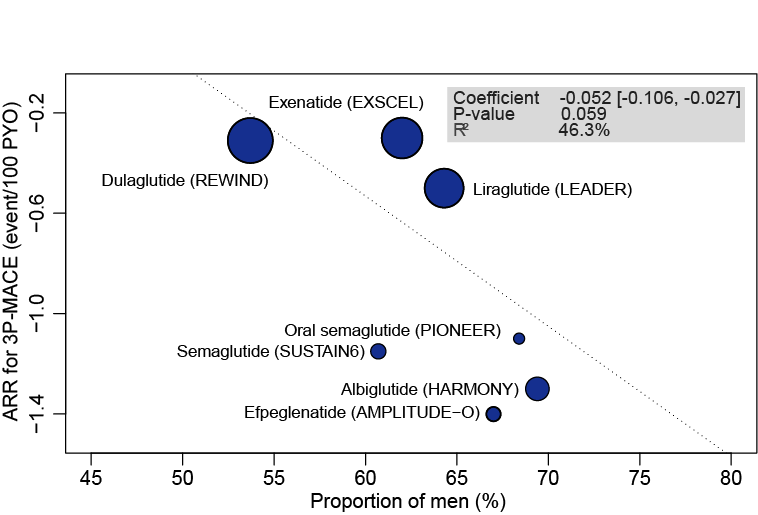 |
| **(C) Efficacy of SGLT-2i and GLP-1RA therapy on ARR for 3P-MACE in men** | **(D) Efficacy of SGLT-2i and GLP-1RA therapy on ARR for 3P-MACE in women** |
| 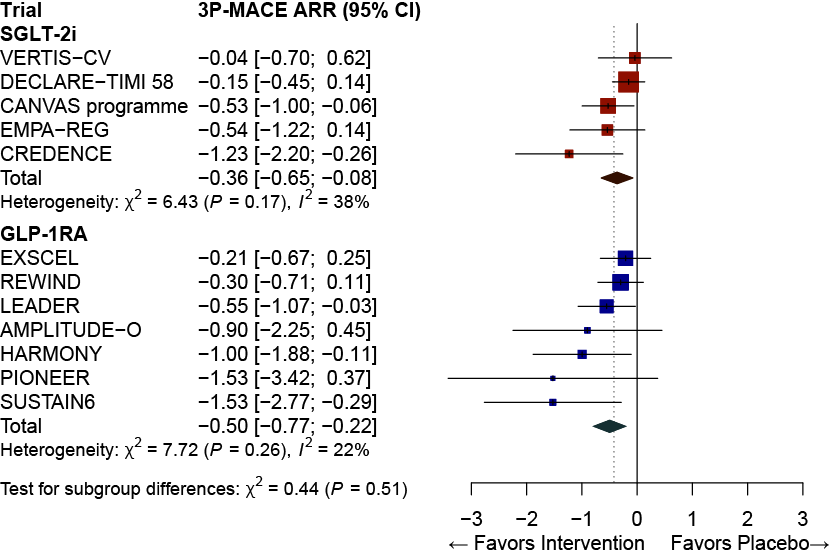 | 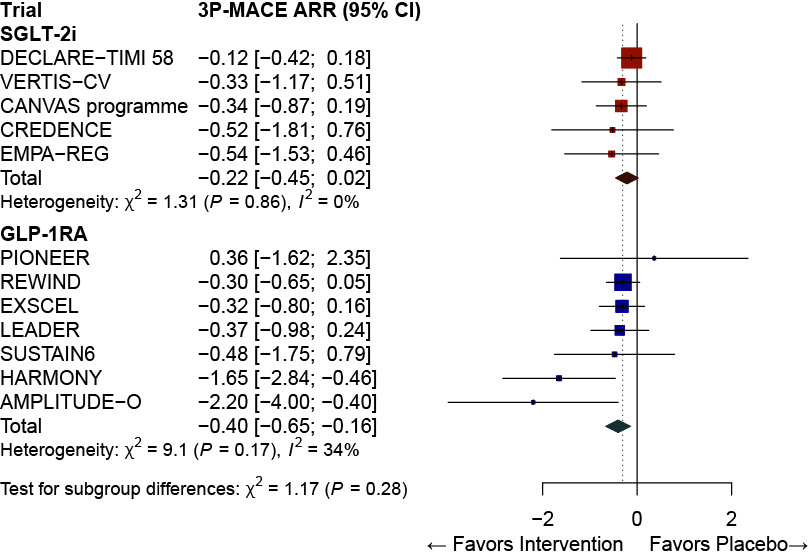 |
| **(E) Meta-regression between RRR for 3P-MACE by SGLT-2i therapy and the proportion of men** | **(F) Meta-regression between RRR for 3P-MACE by GLP-1RA therapy and the proportion of men** |
| 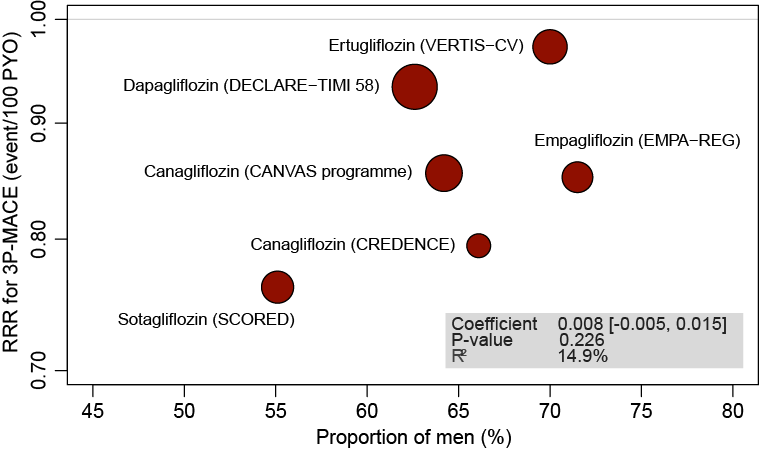 | 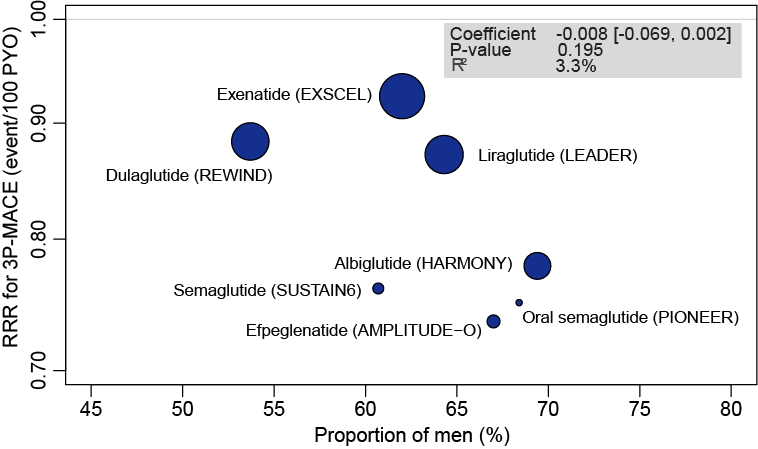 |
| **(G) Efficacy of SGLT-2i and GLP-1RA therapy on RRR for 3P-MACE in men** | **(H) Efficacy of SGLT-2i and GLP-1RA therapy on RRR for 3P-MACE in women** |
| 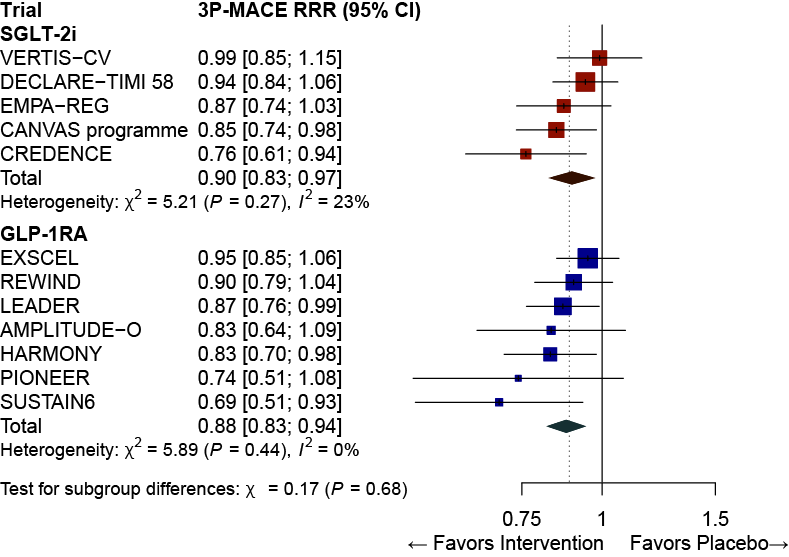 | 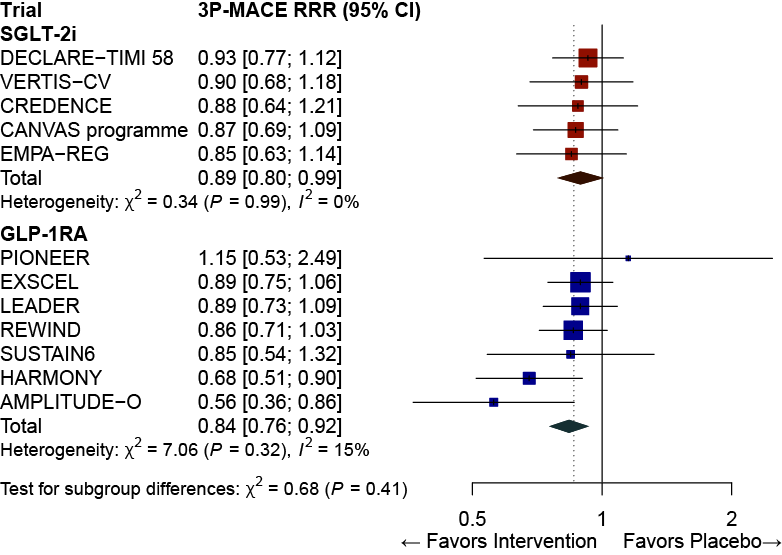 |

**Supplementary Figure S8. Absolute risk reduction (A~D) and Relative risk reduction (E~H) for 3P-MACE by baseline BMI level.** BMI level was divided by 30 kg/m^2^ except for AMPLITUDE-O trial, which used 31.9 kg/m^2^ (the median). The coefficient represents the slope of the regression line. R^2^ indicates the strength of the association of the characteristics. The diamond indicates the pooled estimates, and the boxes are each study with 95% CI. ARR, absolute risk reduction; BMI, body mass index; CI, confidence interval; GLP-1RA, glucagon-like peptide 1 receptor agonists; PYO, person-years of observation; RRR, relative risk reduction; SGLT-2i, sodium-glucose cotransporter-2 inhibitors; 3P-MACE, 3-point major adverse cardiovascular events.

| **(A) Meta-regression between ARR for 3P-MACE by SGLT-2i therapy and average BMI level** | **(B) Meta-regression between ARR for 3P-MACE by GLP-1RA therapy and average BMI level** |
| --- | --- |
| 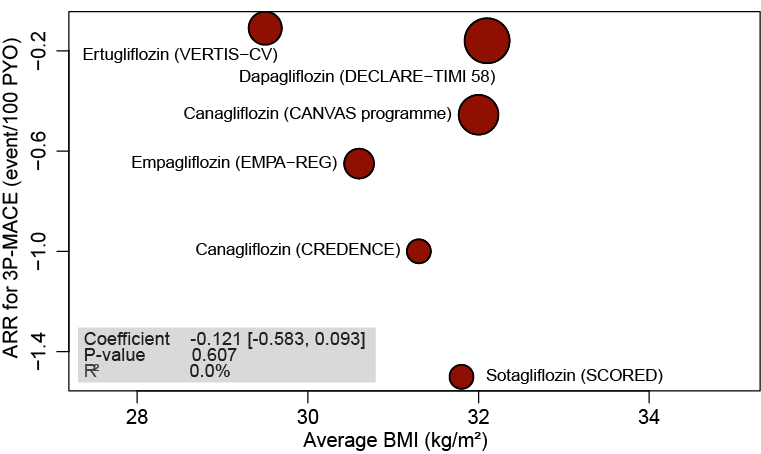 | 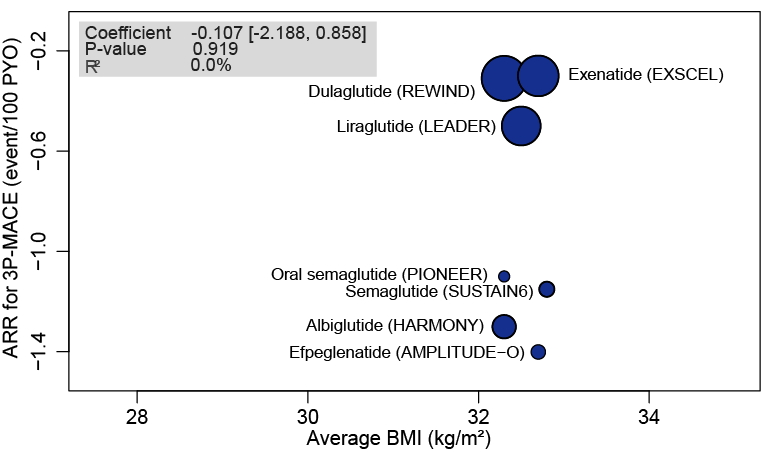 |
| **(C) Efficacy of SGLT-2i and GLP-1RA therapy on ARR for 3P-MACE in the patients with low BMI** | **(D) Efficacy of SGLT-2i and GLP-1RA therapy on ARR for 3P-MACE in the patients with high BMI** |
| 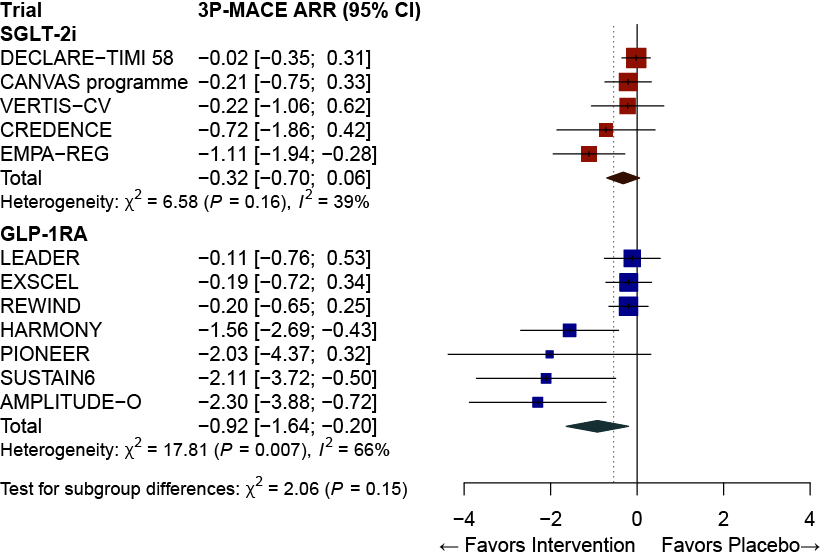 | 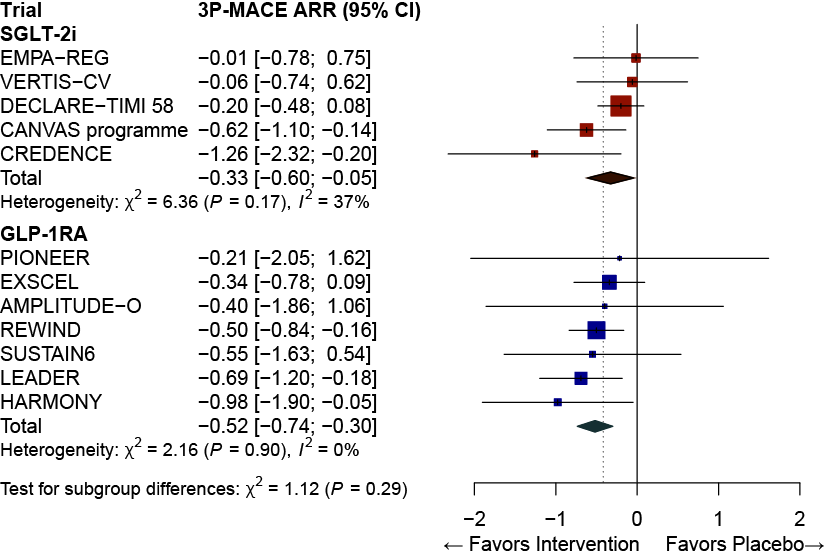 |
| **(E) Meta-regression between RRR for 3P-MACE by SGLT-2i therapy and average BMI level** | **(F) Meta-regression between RRR for 3P-MACE by GLP-1RA therapy and average BMI level** |
| 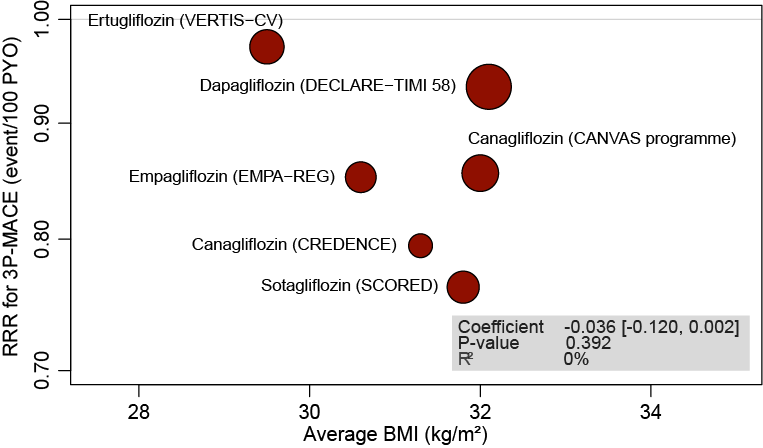 | 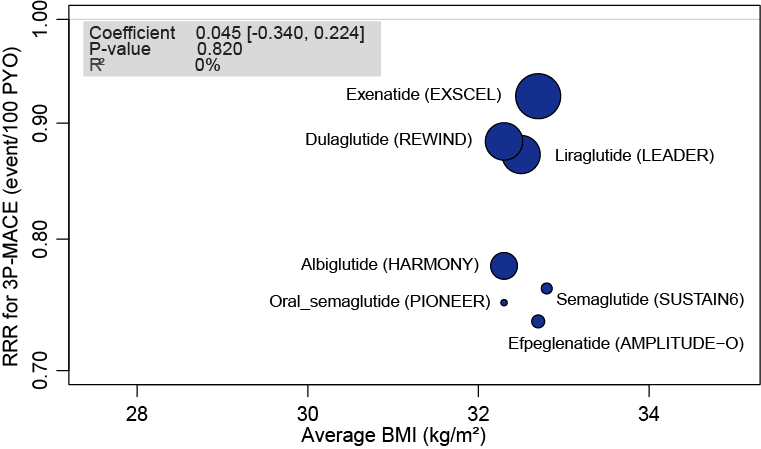 |
| **(G) Efficacy of SGLT-2i and GLP-1RA therapy on RRR for 3P-MACE in the patients with low BMI** | **(H) Efficacy of SGLT-2i and GLP-1RA therapy on RRR for 3P-MACE in the patients with high BMI** |
| 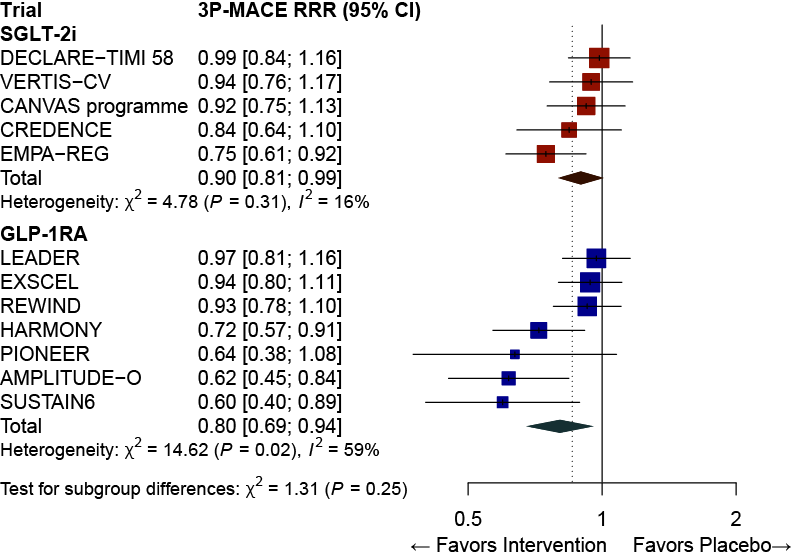 | 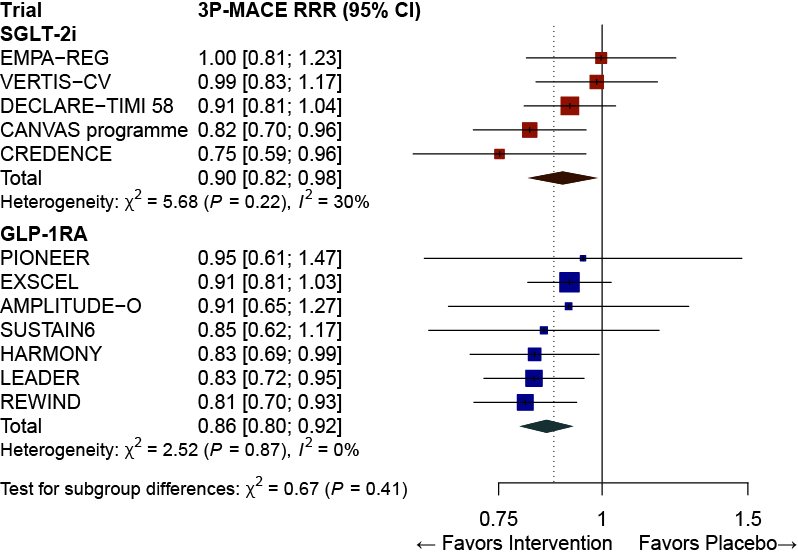 |

**Supplementary Figure S9. Absolute risk reduction (A~D) and Relative risk reduction (E~H) for 3P-MACE by baseline HbA1c level.** Criteria for HbA1c level was different among the trials: most used 8.0%; 8.5% was used in EMPA-REG, VERTIS-CV, SUSTAIN-6, and PIONEER 6; 8.3% in LEADER; and 7.0% in REWIND. The coefficient represents the slope of the regression line. R^2^ indicates the strength of the association of the characteristics. The diamond indicates the pooled estimates, and the boxes are each study with 95% CI. ARR, absolute risk reduction; CI, confidence interval; GLP-1RA, glucagon-like peptide 1 receptor agonists; HbA1c, glycated hemoglobin; PYO, person-years of observation; RRR, relative risk reduction; SGLT-2i, sodium-glucose cotransporter-2 inhibitors; 3P-MACE, 3-point major adverse cardiovascular events.

| **(A) Meta-regression between ARR for 3P-MACE by SGLT-2i therapy and average HbA1c level** | **(B) Meta-regression between ARR for 3P-MACE by GLP-1RA therapy and average HbA1c level** |
| --- | --- |
| 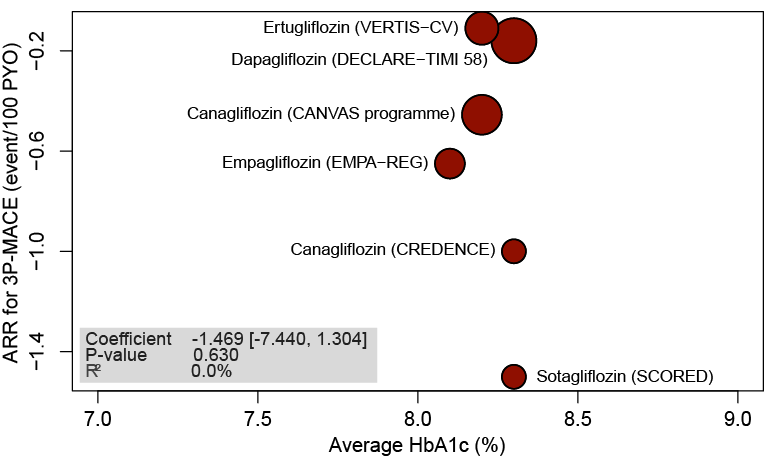 | 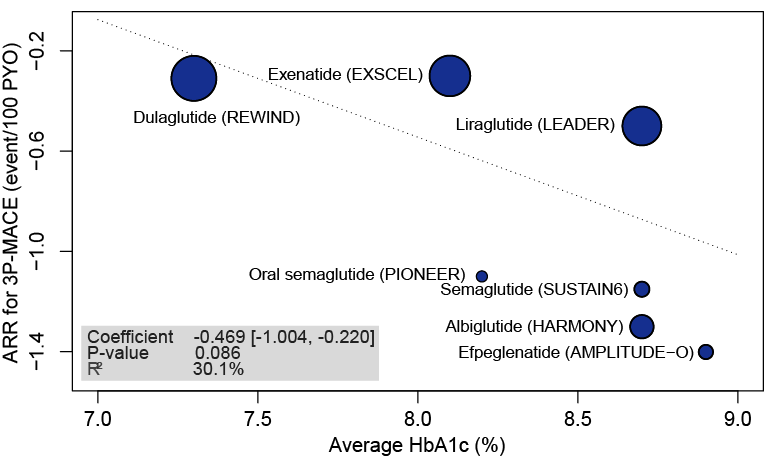 |
| **(C) Efficacy of SGLT-2i and GLP-1RA therapy on ARR for 3P-MACE in the patients with low HbA1c** | **(D) Efficacy of SGLT-2i and GLP-1RA therapy on ARR for 3P-MACE in the patients with high HbA1c** |
| 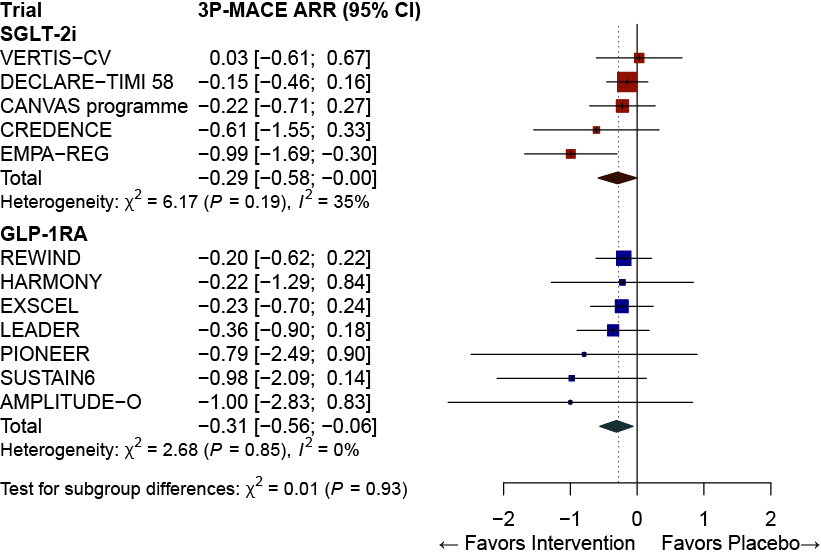 | 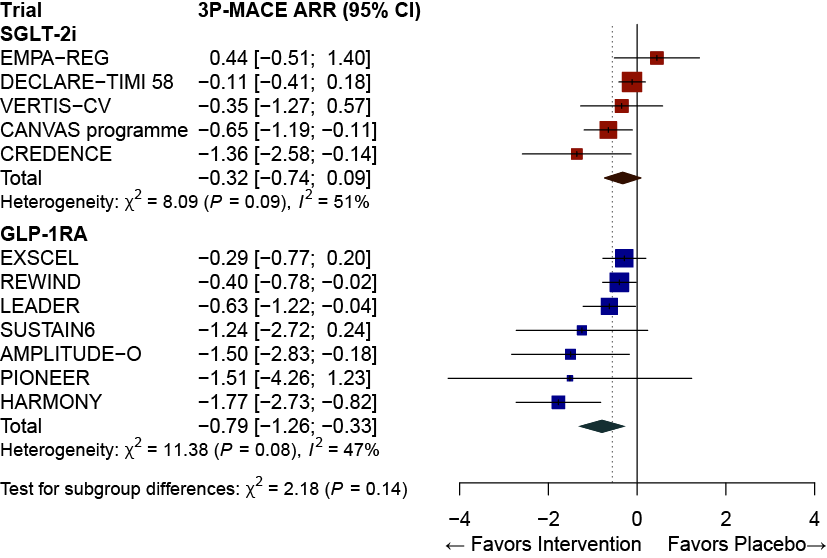 |
| **(E) Meta-regression between RRR for 3P-MACE by SGLT-2i therapy and average HbA1c level** | **(F) Meta-regression between RRR for 3P-MACE by GLP-1RA therapy and average HbA1c level** |
| 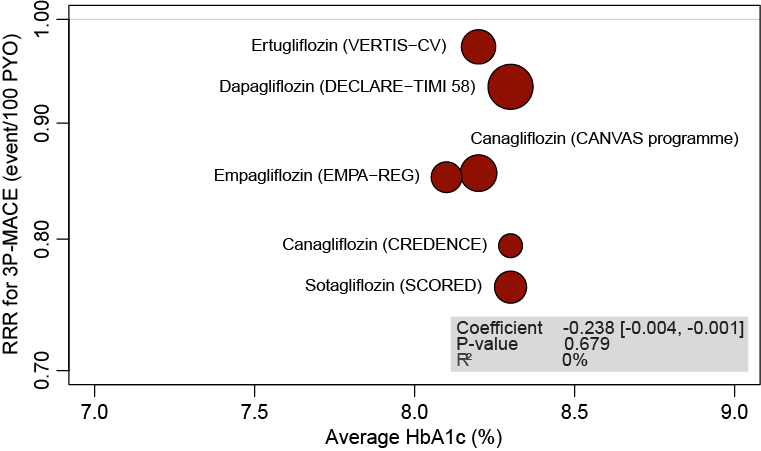 | 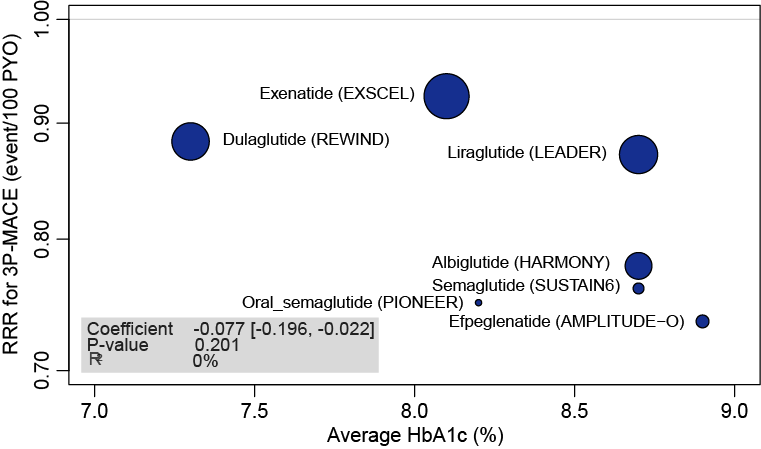 |
| **(G) Efficacy of SGLT-2i and GLP-1RA therapy on RRR for 3P-MACE in the patients with low HbA1c** | **(H) Efficacy of SGLT-2i and GLP-1RA therapy on RRR for 3P-MACE in the patients with high HbA1c** |
| 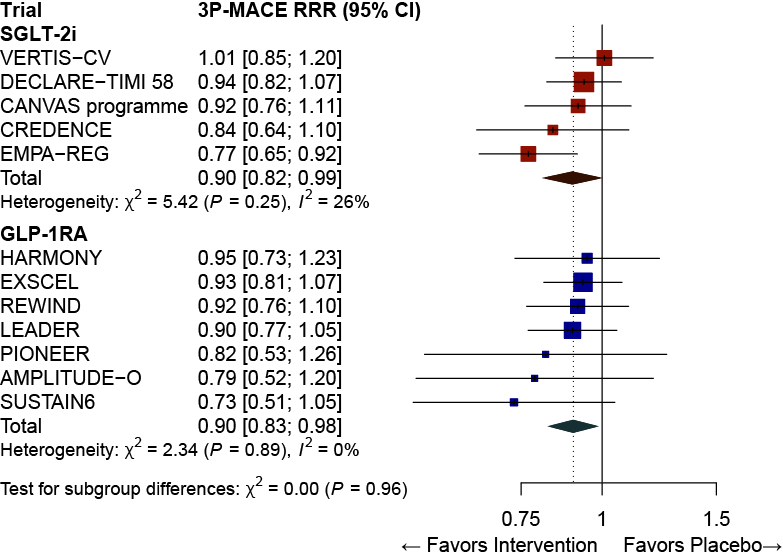 | 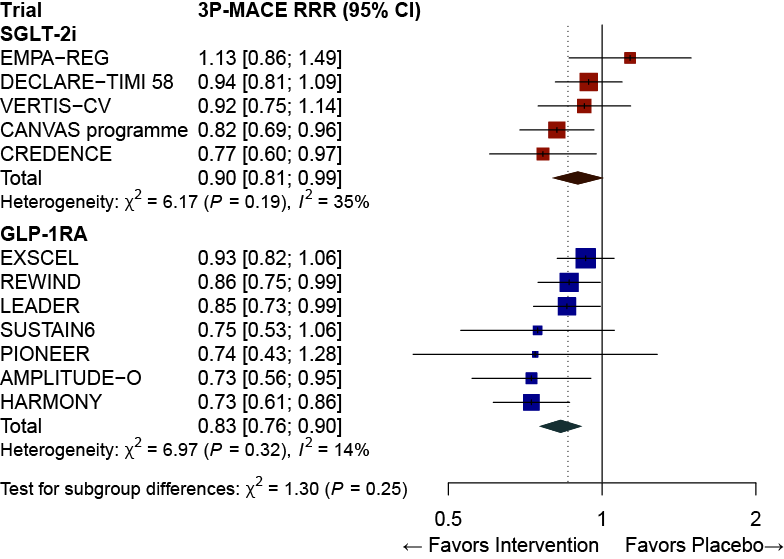 |

**Supplementary Figure S10. Absolute risk reduction (A~D) and Relative risk reduction (E~H) for 3P-MACE by baseline CVD history.** The coefficient represents the slope of the regression line. R^2^ indicates the strength of the association of the characteristics. The diamond indicates the pooled estimates, and the boxes are each study with 95% CI. ARR, absolute risk reduction; CI, confidence interval; CVD, cardiovascular disease; GLP-1RA, glucagon-like peptide 1 receptor agonists; PYO, person-years of observation; RRR, relative risk reduction; SGLT-2i, sodium-glucose cotransporter-2 inhibitors; 3P-MACE, 3-point major adverse cardiovascular events.

| **(A) Meta-regression between ARR for 3P-MACE by SGLT-2i therapy and proportion of patients with previous CVD** | **(B) Meta-regression between ARR for 3P-MACE by GLP-1RA therapy and proportion of patients with previous CVD** | |
| --- | --- | --- |
| 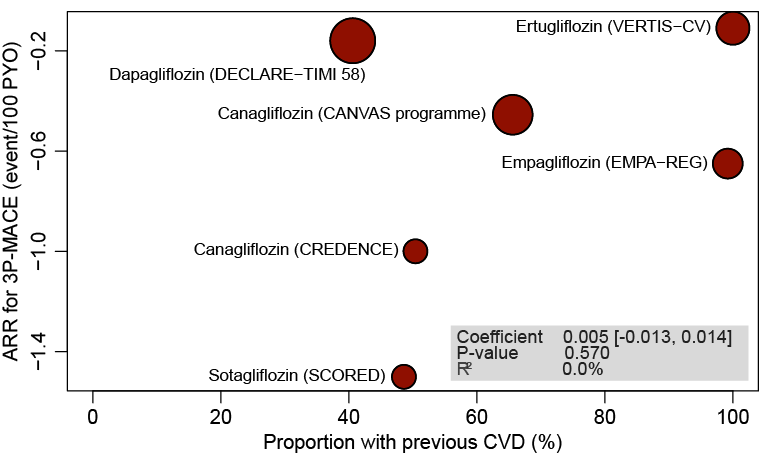 | 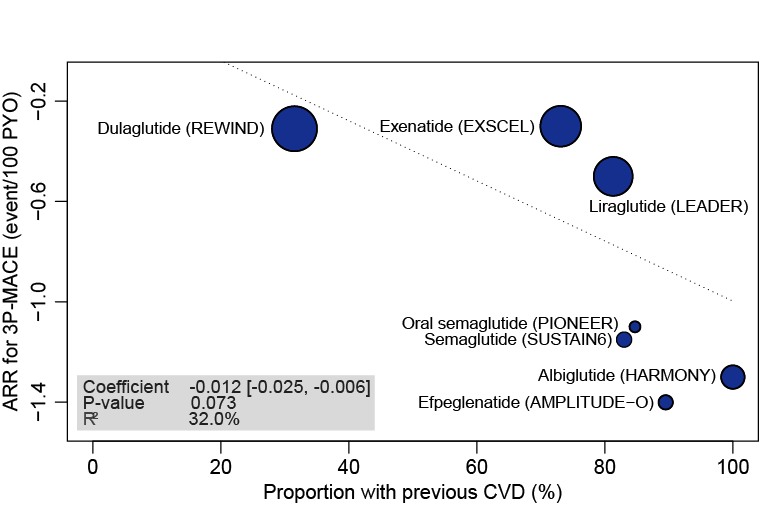 | |
| **(C) Efficacy of SGLT-2i and GLP-1RA therapy on ARR for 3P-MACE without previous CVD** | **(D) Efficacy of SGLT-2i and GLP-1RA therapy on ARR for 3P-MACE with previous CVD** | |
| 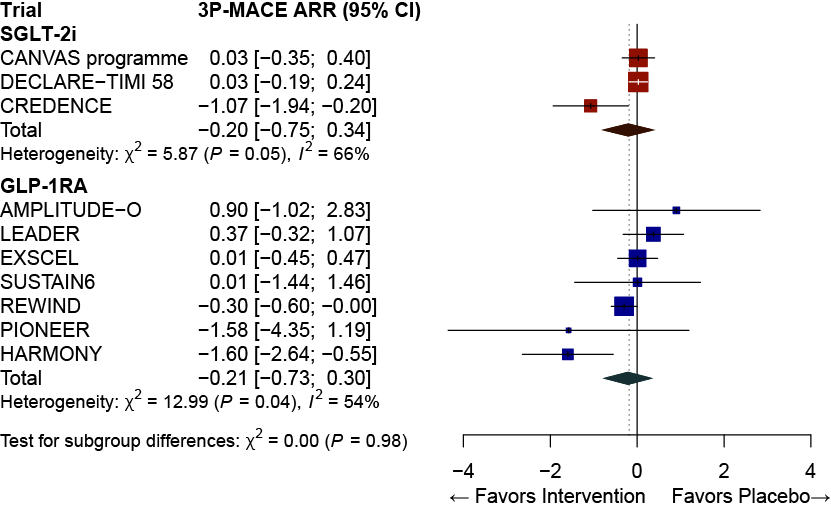 | 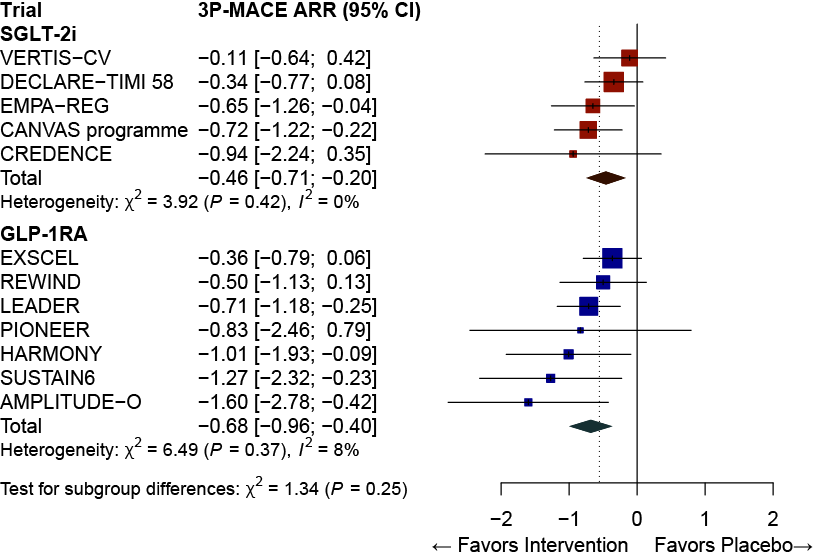 | |
| **(E) Meta-regression between RRR for 3P-MACE by SGLT-2i therapy and the proportion of patients with previous CVD** | | **(F) Meta-regression between RRR for 3P-MACE by GLP-1RA therapy and the proportion of patients with previous CVD** |
| 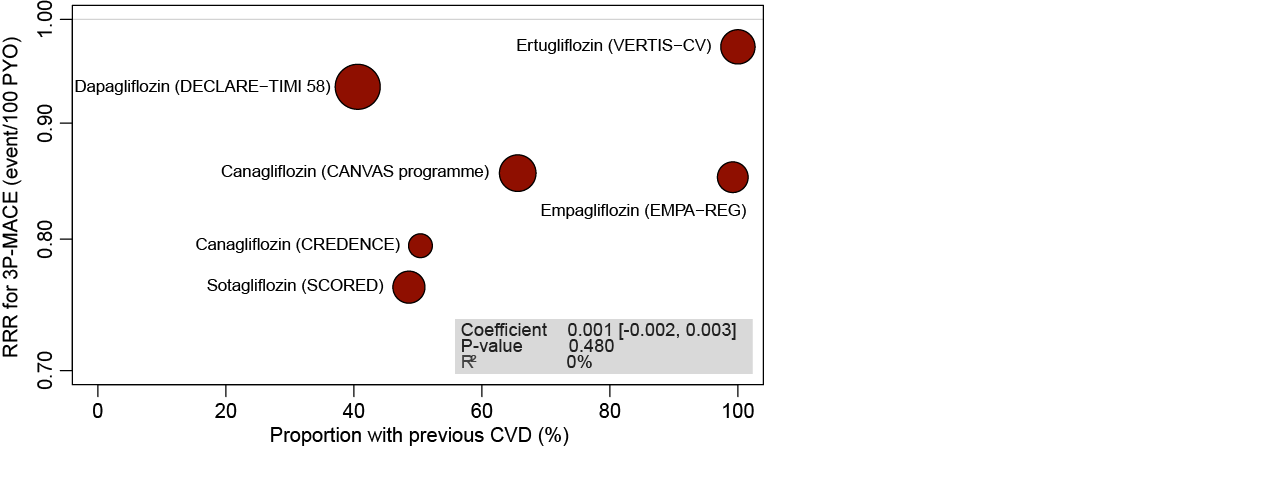 | | 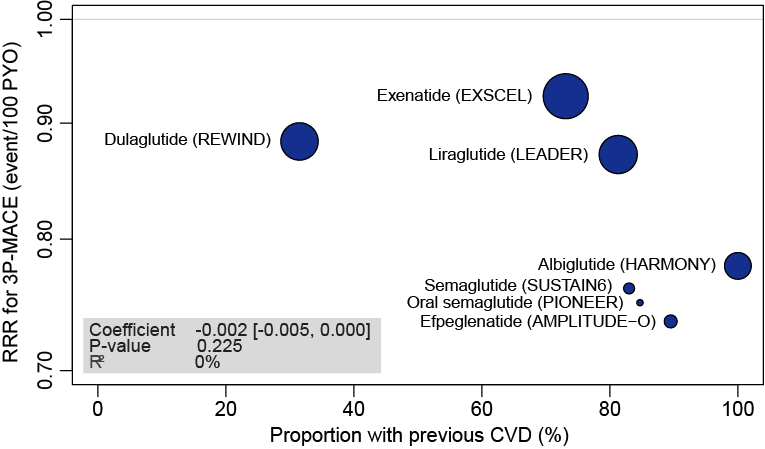 |
| **(G) Efficacy of SGLT-2i and GLP-1RA therapy on RRR for 3P-MACE in the patients without previous CVD** | | **(H) Efficacy of SGLT-2i and GLP-1RA therapy on RRR for 3P-MACE in the patients with previous CVD** |
| 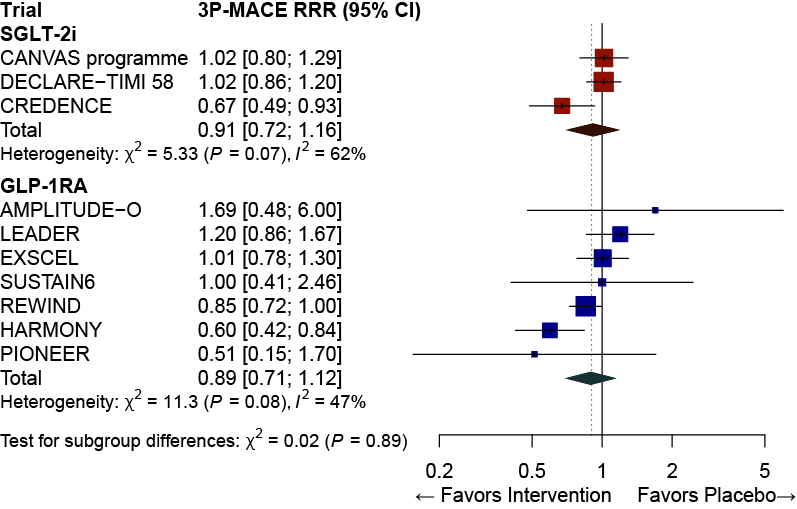 | | 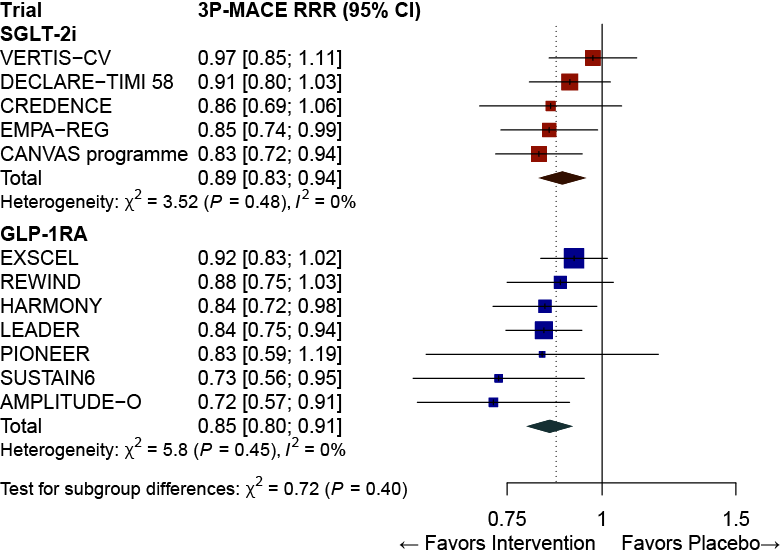 |

**Supplementary Figure S11. Absolute risk reduction (A~D) and Relative risk reduction (E~H) for 3P-MACE by pre-existing HF.** The coefficient represents the slope of the regression line. R^2^ indicates the strength of the association of the characteristics. The diamond indicates the pooled estimates, and the boxes are each study with 95% CI. ARR, absolute risk reduction; CI, confidence interval; GLP-1RA, glucagon-like peptide 1 receptor agonists; HF, heart failure; PYO, person-years of observation; RRR, relative risk reduction; SGLT-2i, sodium-glucose cotransporter-2 inhibitors; 3P-MACE, 3-point major adverse cardiovascular events.

| **(A) Meta-regression between ARR for 3P-MACE by SGLT-2i therapy and the proportion of patients with history of HF** | **(B) Meta-regression between ARR for 3P-MACE by GLP-1RA therapy and the proportion of patients with history of HF** |
| --- | --- |
| 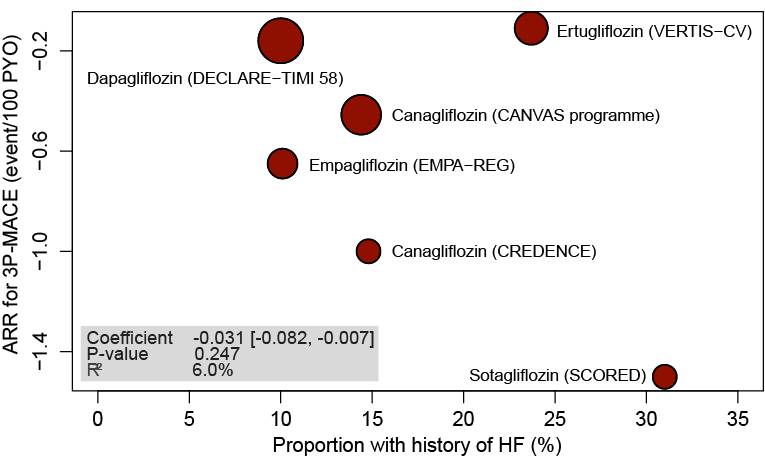 | 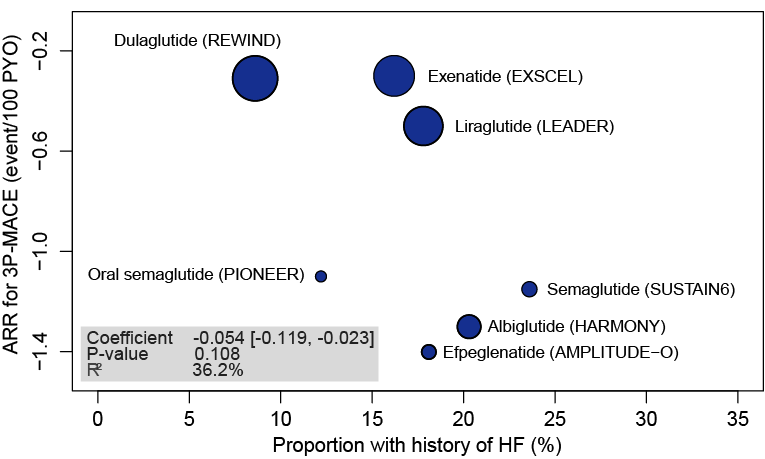 |
| **(C) Efficacy of SGLT-2i and GLP-1RA therapy on ARR for 3P-MACE without HF history** | **(D) Efficacy of SGLT-2i and GLP-1RA therapy on ARR for 3P-MACE with HF history** |
| 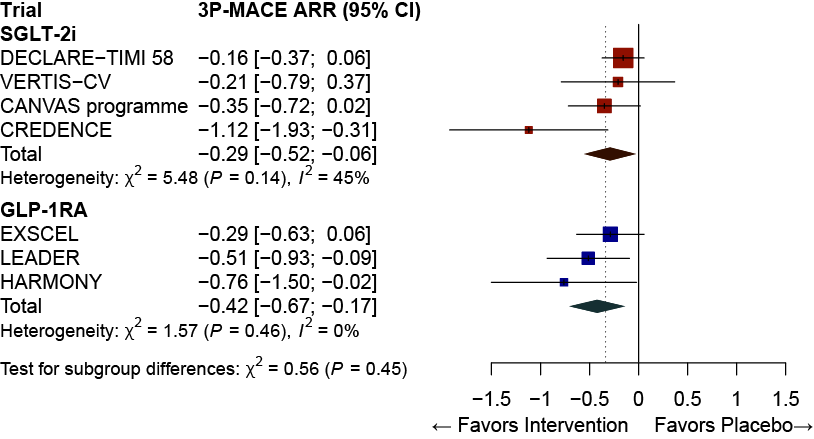 | 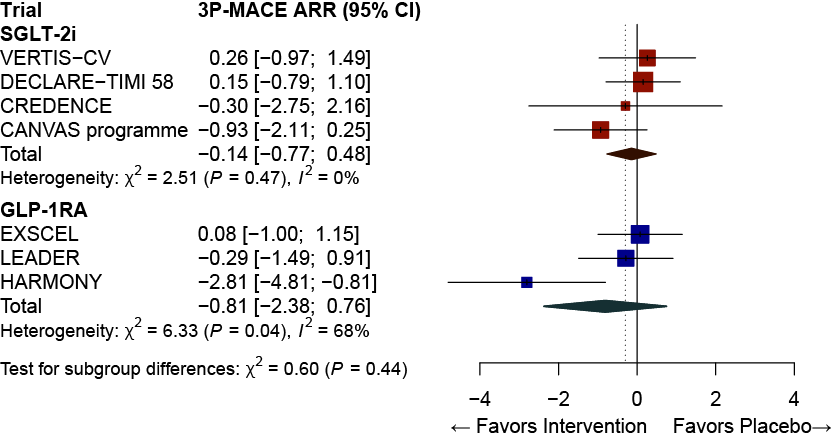 |
| **(E) Meta-regression between RRR for 3P-MACE by SGLT-2i therapy and the proportion of patients with history of HF** | **(F) Meta-regression between RRR for 3P-MACE by GLP-1RA therapy and the proportion of patients with history of HF** |
| 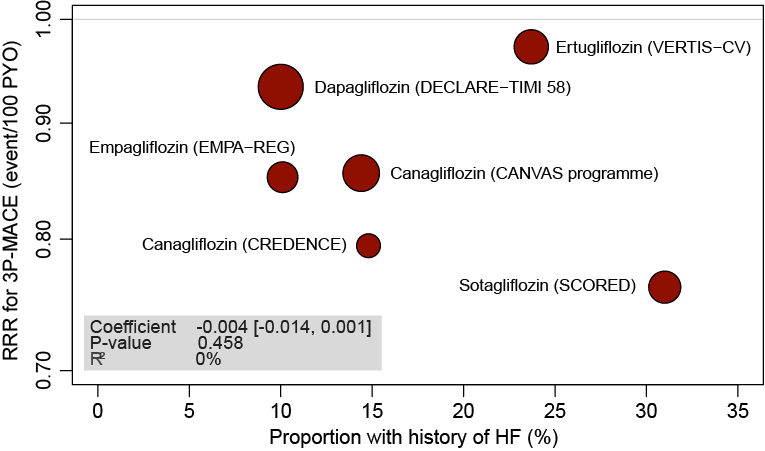 | 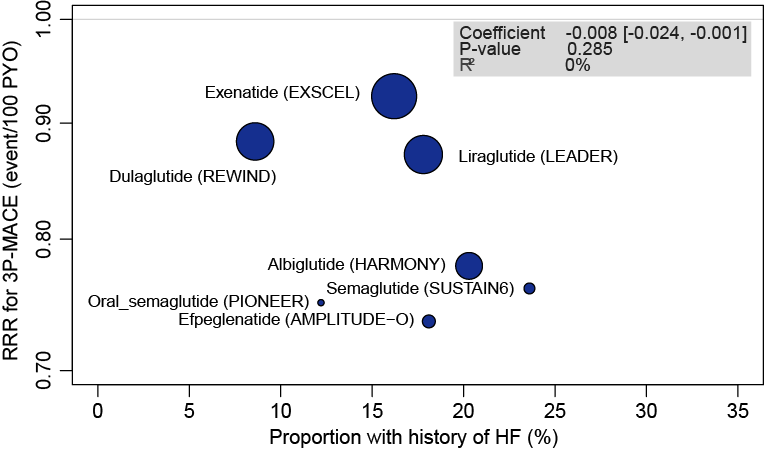 |
| **(G) Efficacy of SGLT-2i and GLP-1RA therapy on RRR for 3P-MACE without HF history** | **(H) Efficacy of SGLT-2i and GLP-1RA therapy on RRR for 3P-MACE with HF history** |
| 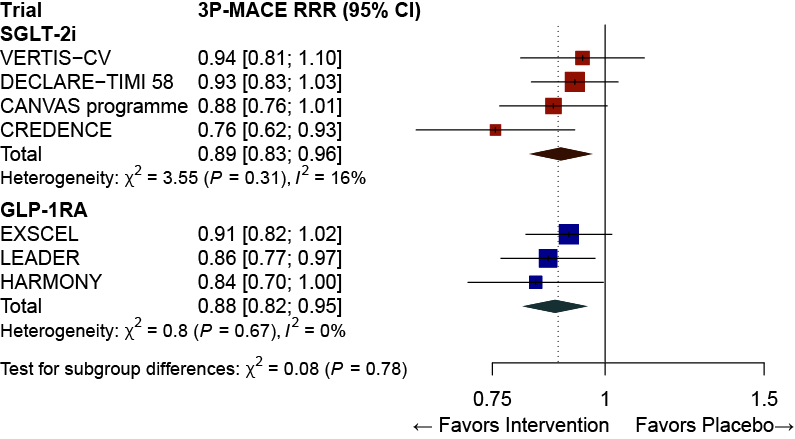 | 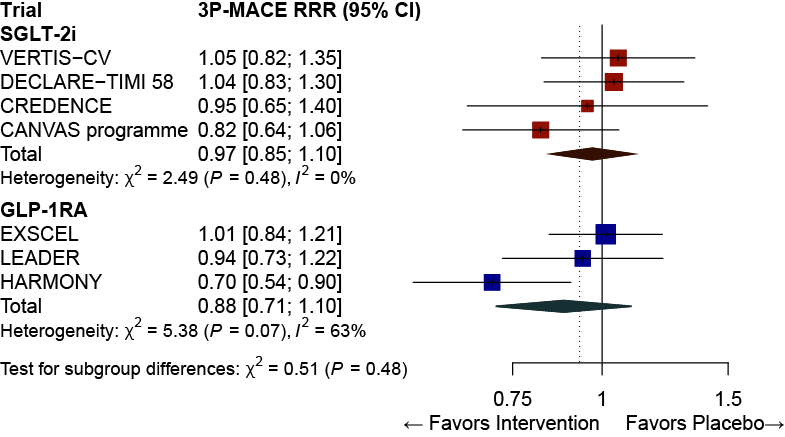 |

**Supplementary Table S1. GRADE evidence profile for the 3-point major adverse cardiovascular events in each subgroup.** ARR, absolute risk reduction; BMI, body mass index; CVD, cardiovascular disease; eGFR, estimated glomerular filtration ratio; GLP-1RA, glucagon-like peptide-1 receptor agonist; HbA1c, glycated hemoglobin; HF, heart failure; RRR, relative risk reduction; SGLT-2i, sodium–glucose cotransporter-2 inhibitor; 3P-MACE, 3-point major adverse cardiovascular events.

| Quality assessment | | | | | | | No of patients | | Effect | | Quality | Importance |
| --- | --- | --- | --- | --- | --- | --- | --- | --- | --- | --- | --- | --- |
| No of studies | Design | Risk of bias | Inconsistency | Indirectness | Imprecision | Other considerations | Outcome | Control | RRR (95% CI) | ARR (95% CI) |  |  |
| Effects on 3P-MACE with overall population by SGLT-2i therapy | | | | | | | | | | | | |
| 6 | randomized trials | not serious | not serious | not serious | not serious | none | 3044/32051 (9.5%) | 2549/25494 (10.0%) | 0.87  (0.81, 0.93) | –0.55  (–0.93, –0.17) | ⊕⊕⊕⊕  High | CRITICAL |
| Effects on 3P-MACE with overall population by GLP-1RA therapy | | | | | | | | | | | | |
| 7 | randomized trials | not serious | not serious | not serious | not serious | none | 2737/27660  (9.9%) | 3037/26352  (11.5%) | 0.85  (0.80, 0.91) | –0.67  (–1.02, –0.32) | ⊕⊕⊕⊕  High | CRITICAL |
| Effects on 3P-MACE in patients with normal eGFR (≥60 mL/min/1.73 m^2^) by SGLT-2i therapy | | | | | | | | | | | | |
| 5 | randomized trials | not serious | not serious | not serious | not serious | none | 1928/21322  (9.0%) | 1529/16076  (9.5%) | 0.93  (0.86, 0.99) | –0.17  (–0.34, –0.01) | ⊕⊕⊕⊕  High | CRITICAL |
| Effects on 3P-MACE in patients with normal eGFR (≥60 mL/min/1.73 m^2^) by GLP-1RA therapy | | | | | | | | | | | | |
| 5 | randomized trials | not serious | serious | not serious | not serious | none | 1311/15283  (8.6%) | 1535/15320  (10.0%) | 0.83  (0.74, 0.94) | –0.68  (–1.19, –0.17) | ⊕⊕⊕◯  Moderate | CRITICAL |
| Effects on 3P-MACE in patients with reduced eGFR (<60 mL/min/1.73 m^2^) by SGLT-2i therapy | | | | | | | | | | | | |
| 6 | randomized trials | not serious | not serious | not serious | not serious | none | 1077/10726  (10.0%) | 1083/9417  (11.5%) | 0.83  (0.74, 0.92) | –0.90  (–1.44, –0.37) | ⊕⊕⊕⊕  High | CRITICAL |
| Effects on 3P-MACE in patients with reduced eGFR (<60 mL/min/1.73 m^2^) by GLP-1RA therapy | | | | | | | | | | | | |
| 5 | randomized trials | not serious | not serious | not serious | not serious | none | 636/4682  (13.6%) | 713/4684  (15.2%) | 0.88  (0.74, 1.04) | –0.71  (–1.59, 0.17) | ⊕⊕⊕⊕  High | CRITICAL |
| Effects on 3P-MACE in patients with normoalbuminuria by SGLT-2i therapy | | | | | | | | | | | | |
| 5 | randomized trials | not serious | not serious | not serious | not serious | none | 1406/17113  (8.2%) | 1185/14074  (8.4%) | 0.94  (0.87, 1.02) | –0.16  (–0.38, 0.06) | ⊕⊕⊕⊕  High | CRITICAL |
| Effects on 3P-MACE in patients with normoalbuminuria by GLP-1RA therapy | | | | | | | | | | | | |
| 1 | randomized trials | not serious | not serious | not serious | very serious | none | 318/4668  (6.8%) | 335/4672  (7.2%) | 0.94  (0.80, 1.09) | –0.19  (–0.63, 0.25) | ⊕⊕◯◯  Low | CRITICAL |
| Effects on 3P-MACE in patients with albuminuria (≥30 mg/g) by SGLT-2i therapy | | | | | | | | | | | | |
| 6 | randomized trials | not serious | serious | not serious | not serious | none | 1621/13588  (11.9%) | 1582/11588  (13.7%) | 0.84  (0.75, 0.94) | –0.89  (–1.71, –0.08) | ⊕⊕⊕◯  Moderate | CRITICAL |
| Effects on 3P-MACE in patients with albuminuria (≥30 mg/g) by GLP-1RA therapy | | | | | | | | | | | | |
| 1 | randomized trials | not serious | not serious | not serious | very serious | none | 277/1684  (16.4%) | 338/1728  (19.6%) | 0.82  (0.72, 0.99) | –0.82  (–1.57, –0.07) | ⊕⊕◯◯  Low | CRITICAL |
| Effects on 3P-MACE in patients with younger age group by SGLT-2i therapy | | | | | | | | | | | | |
| 5 | randomized trials | not serious | not serious | not serious | not serious | none | 1199/14033  (8.5%) | 885/9792  (9.0%) | 0.92  (0.85, 1.01) | –0.16  (–0.37, 0.05) | ⊕⊕⊕⊕  High | CRITICAL |
| Effects on 3P-MACE in patients with younger age group by GLP-1RA therapy | | | | | | | | | | | | |
| 7 | randomized trials | not serious | serious | not serious | not serious | none | 1108/13445 (8.2%) | 1191/12627 (9.4%) | 0.82  (0.70, 0.95) | –0.73  (–1.32, –0.13) | ⊕⊕⊕◯  Moderate | CRITICAL |
| Effects on 3P-MACE in patients with older age group by SGLT-2i therapy | | | | | | | | | | | | |
| 5 | randomized trials | not serious | serious | not serious | not serious | none | 1499/12726 (11.8%) | 1367/10410 (13.1%) | 0.87  (0.78, 0.97) | –0.54  (–1.04, –0.05) | ⊕⊕⊕◯  Moderate | CRITICAL |
| Effects on 3P-MACE in patients with older age group by GLP-1RA therapy | | | | | | | | | | | | |
| 7 | randomized trials | not serious | not serious | not serious | not serious | none | 1629/14215 (11.5%) | 1846/13725 (13.4%) | 0.86  (0.81, 0.92) | –0.55  (–0.81, –0.28) | ⊕⊕⊕⊕  High | CRITICAL |
| Effects on 3P-MACE in men group by SGLT-2i therapy | | | | | | | | | | | | |
| 5 | randomized trials | not serious | not serious | not serious | not serious | none | 1979/17809 (11.1%) | 1575/13125 (12.0%) | 0.90  (0.83, 0.97) | –0.36  (–0.65, –0.08) | ⊕⊕⊕⊕  High | CRITICAL |
| Effects on 3P-MACE in men group by GLP-1RA therapy | | | | | | | | | | | | |
| 7 | randomized trials | not serious | not serious | not serious | not serious | none | 1921/17409 (11.0%) | 2096/16534 (12.7%) | 0.88  (0.83, 0.94) | –0.50  (–0.77, –0.22) | ⊕⊕⊕⊕  High | CRITICAL |
| Effects on 3P-MACE in women by SGLT-2i therapy | | | | | | | | | | | | |
| 5 | randomized trials | not serious | not serious | not serious | not serious | none | 699/8950 (7.8%) | 597/7077 (8.4%) | 0.89  (0.80, 0.99) | –0.22  (–0.45, 0.02) | ⊕⊕⊕⊕  High | CRITICAL |
| Effects on 3P-MACE in women by GLP-1RA therapy | | | | | | | | | | | | |
| 7 | randomized trials | not serious | not serious | not serious | not serious | none | 816/10251 (8.0%) | 941/9818 (9.6%) | 0.84  (0.76, 0.92) | –0.40  (–0.65, –0.16) | ⊕⊕⊕⊕  High | CRITICAL |
| Effects on 3P-MACE in patients with low BMI by SGLT-2i therapy | | | | | | | | | | | | |
| 5 | randomized trials | not serious | not serious | not serious | not serious | none | 1044/10809 (9.7%) | 895/8687 (10.3%) | 0.90  (0.81, 0.99) | –0.32  (–0.70, 0.06) | ⊕⊕⊕⊕  High | CRITICAL |
| Effects on 3P-MACE in patients with low BMI by GLP-1RA therapy | | | | | | | | | | | | |
| 7 | randomized trials | not serious | serious | not serious | not serious | none | 1022/10681 (9.6%) | 1156/10196 (11.3%) | 0.80  (0.69, 0.94) | –0.92  (–1.64, –0.20) | ⊕⊕⊕◯  Moderate | CRITICAL |
| Effects on 3P-MACE in patients with high BMI by SGLT-2i therapy | | | | | | | | | | | | |
| 5 | randomized trials | not serious | not serious | not serious | not serious | none | 1537/14962 (10.3%) | 1324/11942 (11.1%) | 0.90  (0.82, 0.98) | –0.33  (–0.60, –0.05) | ⊕⊕⊕⊕  High | CRITICAL |
| Effects on 3P-MACE in patients with high BMI by GLP-1RA therapy | | | | | | | | | | | | |
| 7 | randomized trials | not serious | not serious | not serious | not serious | none | 1694/16868 (10.0%) | 1865/16046 (11.6%) | 0.86  (0.80, 0.92) | –0.52  (–0.74, –0.30) | ⊕⊕⊕⊕  High | CRITICAL |
| Effects on 3P-MACE in patients with low HbA1c by SGLT-2i therapy | | | | | | | | | | | | |
| 5 | randomized trials | not serious | not serious | not serious | not serious | none | 1499/15122 (9.9%) | 1153/10735 (10.7%) | 0.90  (0.82, 0.99) | –0.31  (–0.63, 0.01) | ⊕⊕⊕⊕  High | CRITICAL |
| Effects on 3P-MACE in patients with low HbA1c by GLP-1RA therapy | | | | | | | | | | | | |
| 7 | randomized trials | not serious | not serious | not serious | not serious | none | 1158/12476 (9.3%) | 1267/12196 (10.4%) | 0.90  (0.83, 0.98) | –0.31  (–0.56, –0.06) | ⊕⊕⊕⊕  High | CRITICAL |
| Effects on 3P-MACE in patients with high HbA1c by SGLT-2i therapy | | | | | | | | | | | | |
| 5 | randomized trials | not serious | serious | not serious | not serious | none | 1198/11885 (10.1%) | 950/8899 (10.7%) | 0.90  (0.82, 1.00) | –0.28  (–0.67, 0.11) | ⊕⊕⊕◯  Moderate | CRITICAL |
| Effects on 3P-MACE in patients with high HbA1c by GLP-1RA therapy | | | | | | | | | | | | |
| 7 | randomized trials | not serious | not serious | not serious | not serious | none | 1570/15121 (10.4%) | 1766/14089 (12.5%) | 0.83  (0.76, 0.90) | –0.79  (–1.26, –0.33) | ⊕⊕⊕⊕  High | CRITICAL |
| Effects on 3P-MACE in patients without previous CVD by SGLT-2i therapy | | | | | | | | | | | | |
| 3 | randomized trials | not serious | serious | not serious | serious | none | 451/8236 (5.5%) | 519/9065 (5.7%) | 0.91  (0.72, 1.16) | –0.20  (–0.75, 0.34) | ⊕⊕◯◯  Low | CRITICAL |
| Effects on 3P-MACE in patients without previous CVD by GLP-1RA therapy | | | | | | | | | | | | |
| 7 | randomized trials | not serious | serious | not serious | not serious | none | 545/8123 (6.7%) | 609/8071 (7.5%) | 0.89  (0.71, 1.12) | –0.21  (–0.73, 0.30) | ⊕⊕⊕◯  Moderate | CRITICAL |
| Effects on 3P-MACE in patients with previous CVD by SGLT-2i therapy | | | | | | | | | | | | |
| 5 | randomized trials | not serious | not serious | not serious | not serious | none | 2242/18523 (12.1%) | 1755/12585 (13.9%) | 0.89  (0.83, 0.94) | –0.44  (–0.67, –0.21) | ⊕⊕⊕⊕  High | CRITICAL |
| Effects on 3P-MACE in patients with previous CVD by GLP-1RA therapy | | | | | | | | | | | | |
| 7 | randomized trials | not serious | not serious | not serious | not serious | none | 2155/19241 (11.2%) | 2397/18011 (13.3%) | 0.85  (0.80, 0.91) | –0.68  (–0.96, –0.40) | ⊕⊕⊕⊕  High | CRITICAL |
| Effects on 3P-MACE in patients without HF history by SGLT-2i therapy | | | | | | | | | | | | |
| 4 | randomized trials | not serious | not serious | not serious | not serious | none | 1675/18802 (8.9%) | 1478/15345 (9.6%) | 0.89  (0.83, 0.96) | –0.29  (–0.52, –0.06) | ⊕⊕⊕⊕  High | CRITICAL |
| Effects on 3P-MACE in patients without HF history by GLP-1RA therapy | | | | | | | | | | | | |
| 3 | randomized trials | not serious | not serious | not serious | serious | none | 1345/13986 (9.6%) | 1525/13952 (10.9%) | 0.88  (0.82, 0.95) | –0.42  (–0.67, –0.17) | ⊕⊕⊕◯  Moderate | CRITICAL |
| Effects on 3P-MACE in patients with HF history by SGLT-2i therapy | | | | | | | | | | | | |
| 4 | randomized trials | not serious | not serious | not serious | not serious | none | 519/3270 (15.9%) | 419/2524 (16.6%) | 0.97  (0.85, 1.10) | –0.14  (–0.77, 0.48) | ⊕⊕⊕⊕  High | CRITICAL |
| Effects on 3P-MACE in patients with HF history by GLP-1RA therapy | | | | | | | | | | | | |
| 3 | randomized trials | not serious | serious | not serious | serious | none | 440/2768 (15.9%) | 502/2848 (17.6%) | 0.88  (0.71, 1.10) | –0.81  (–2.38, 0.76) | ⊕⊕◯◯  Low | CRITICAL |
